# Comparative genomics reveals the distinct evolutionary trajectories of the robust and complex coral lineages

**DOI:** 10.1186/s13059-018-1552-8

**Published:** 2018-11-02

**Authors:** Hua Ying, Ira Cooke, Susanne Sprungala, Weiwen Wang, David C. Hayward, Yurong Tang, Gavin Huttley, Eldon E. Ball, Sylvain Forêt, David J. Miller

**Affiliations:** 1 Division of Ecology and Evolution, Research School of Biology, Australian National University, Acton, ACT 2601 Australia; 2 Comparative Genomics Centre, Department of Molecular and Cell Biology, James Cook University, Townsville, QLD 4811 Australia; 3 Computational Biology and Bioinformatics Unit, Research School of Biology, Australian National University, Acton, ACT 2601 Australia; 4 ARC Centre of Excellence for Coral Reef Studies, James Cook University, Townsville, QLD 4811 Australia

**Keywords:** Scleractinia, Complex coral, Robust coral, Nucleotide substitution model, Hox cluster, ParaHox, Gene family expansion, Histidine biosynthesis

## Abstract

**Background:**

Despite the biological and economic significance of scleractinian reef-building corals, the lack of large molecular datasets for a representative range of species limits understanding of many aspects of their biology. Within the Scleractinia, based on molecular evidence, it is generally recognised that there are two major clades, Complexa and Robusta, but the genomic bases of significant differences between them remain unclear.

**Results:**

Draft genome assemblies and annotations were generated for three coral species: *Galaxea fascicularis* (Complexa), *Fungia sp.*, and *Goniastrea aspera* (Robusta). Whilst phylogenetic analyses strongly support a deep split between Complexa and Robusta, synteny analyses reveal a high level of gene order conservation between all corals, but not between corals and sea anemones or between sea anemones. HOX-related gene clusters are, however, well preserved across all of these combinations. Differences between species are apparent in the distribution and numbers of protein domains and an apparent correlation between number of HSP20 proteins and stress tolerance. Uniquely amongst animals, a complete histidine biosynthesis pathway is present in robust corals but not in complex corals or sea anemones. This pathway appears to be ancestral, and its retention in the robust coral lineage has important implications for coral nutrition and symbiosis.

**Conclusions:**

The availability of three new coral genomes enabled recognition of a de novo histidine biosynthesis pathway in robust corals which is only the second identified biosynthetic difference between corals. These datasets provide a platform for understanding many aspects of coral biology, particularly the interactions of corals with their endosymbionts.

**Electronic supplementary material:**

The online version of this article (10.1186/s13059-018-1552-8) contains supplementary material, which is available to authorized users.

## Background

Despite their ecological and economic significance, many aspects of the biology of the reef-building corals (anthozoan cnidarians belonging to the order Scleractinia) are poorly understood. The calcified Scleractinia made a dramatic appearance in the fossil record in the mid-Triassic (~ 240 MYA), but by this stage they were already morphologically diverse, implying a much earlier origin for the order [1–4]. Classical coral taxonomy relied heavily on a small number of morphological features, but molecular data often contradict groupings based on these traditional criteria. For example, many traditionally defined coral families were para- (or sometimes poly-) phyletic in molecular analyses [5, 6].

Although the timing of the origins and major divergences within the Scleractinia remains equivocal, all of the available molecular data imply that most extant corals fall into two major clades (“superfamilies”) known as the Complexa (complex corals) and Robusta (robust corals). This dichotomy was originally proposed based on partial 16S rDNA data [7] and is supported in the majority of molecular analyses [8–10]. The nomenclature (Complexa/Robusta) was chosen to reflect perceived differences in extent/density of calcification in the range of corals originally studied, “complex” corals being nominally less heavily calcified than “robust” corals [7]. Although this generalisation is questionable, the Complexa/Robusta nomenclature still stands and the split is recognised as real, despite the fact that few morphological or biological criteria resolve the two groups.

One characteristic by which robust and complex corals can be distinguished is mitochondrial genome composition. The mt genomes of robust corals have significantly lower (G + C) content than those of complex corals or corallimorpharians, one consequence of which appears to be significantly higher phenylalanine content in mitochondrially encoded proteins [11]. It has been speculated that these differences might reflect impaired mtDNA repair in robust corals [11], but empirical data in support of this are as yet lacking. Also, based on a limited number of species, differences appear to exist in the early development of robust and complex corals [12]. Amongst corals, early development has been most extensively studied in *Acropora* (a complex coral) species, where gastrulation occurs from what is colloquially known as a “prawn chip”—essentially a bilayer of undifferentiated cells that lacks a blastocoel [13, 14]. Similar developmental patterns have been documented in a number of other complex corals, but not in robust corals, where gastrulation occurs by invagination of an essentially spherical blastula [12, 15, 16].

One reason for the lack of features distinguishing the two clades is the relative paucity of large molecular datasets for a representative range of corals. Until recently, whole genome data have been available for only two anthozoan cnidarians—the (complex) coral *Acropora digitifera* [17], which has endosymbiotic *Symbiodinium*, and the sea anemone *Nematostella vectensis* [18], which lacks them. More recently, genome assemblies for two other anthozoans which harbour endosymbiotic *Symbiodinium* have become available; those of the sea anemone *Aiptasia* [19] and the robust coral *Stylophora pistillata* [20]. The availability of the latter assembly permitted the first whole-genome comparisons to be made between robust and complex corals [20]. Note that in the present paper we have retained the usage “*Aiptasia*”, which was used by Baumgarten et al. [19], due to taxonomic uncertainty. Where coral genera are mentioned without an explicit statement of clade, a (C) or an (R) has been placed after the name of the genus or species, as appropriate.

To provide a platform for investigation of both differences between individual species and the broader question of general differences between complex and robust corals, genome sequencing and assembly was carried out on a number of corals selected to reflect phenotypic and physiological diversity [21].

To broaden the range of species for which data are available, here we report the assembly of the genomes of two robust corals, *Goniastrea aspera* (also known as *Coelastrea aspera*, NCBI:txid1540031) and *Fungia sp.* (NCBI:txid46712), and the complex coral *Galaxea fascicularis* (NCBI:txid46745). *Goniastrea* (R) and *Galaxea* (C) are both regarded as “massive” species, whereas *Fungia* (R) is a solitary coral (a single very large polyp, rather than a colony of smaller individual polyps). Whilst all three have widespread distribution ranges throughout the Indo-Pacific and occur in relatively shallow water, *Goniastrea* (R) is regarded as one of the most environmentally tolerant species on Indo-Pacific reefs [22], frequently dominating intertidal zones where it endures prolonged exposure. Indeed, Veron [23] has described it as being “encountered frequently in places where no coral might be expected to live”. The stress tolerance of *Goniastrea* is in marked contrast to the sensitivity of the two branching corals *Acropora digitifera* (C) and *Stylophora pistillata* (R) [24] for which genome data are available [17, 20]. Whilst all of these species harbour the photosynthetic endosymbiont *Symbiodinium*, heterotrophy is thought to play a major role in *Galaxea* (C) nutrition [25] and this species is atypical in that its polyps are frequently extended for feeding during the day. Other biological characteristics of these species are summarised in Additional file 1: Table S1.

The present study makes genome-wide comparisons amongst eight species of anthozoan cnidarians, of which four are complex corals, two are robust corals and two are sea anemones. It provides the strongest support available to date for the robust/complex split due to application of the non-stationary general Markov nucleotide substitution model which, at such time depth, is particularly significant. Synteny analyses indicated a remarkable degree of gene order conservation between all corals, but only limited conservation between corals and sea anemones. An exception to this is a cluster of homeobox genes, the order of which is conserved not only between complex and robust corals, but also between corals and the sea anemone, *Nematostella*. Coral species differed significantly in terms of PFAM-A domain numbers and distribution, and a correlation between stress tolerance and numbers of HSP20/α-crystallin domains was tentatively identified. The most surprising difference, however, was the presence of a fungal-like histidine biosynthesis pathway in robust corals, which is not present in complex corals or sea anemones. This pathway appears to be ancestral and assuming that it is functional, its retention in the robust coral lineage has important implications for coral nutrition and symbiosis.

## Results and discussion

### Genome assembly and annotation

**Fig. 1:**
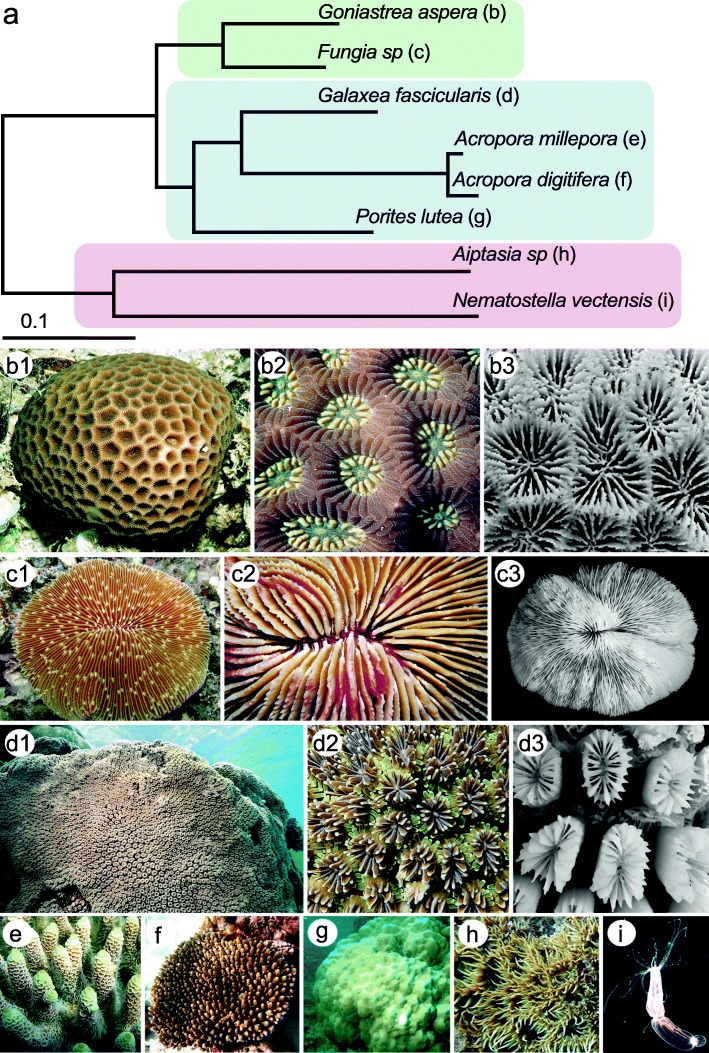
Phylogenetic positions and morphology of cnidarians used in the present study. **a** Molecular phylogeny of corals and sea anemones inferred using a maximum likelihood method based on the general nucleotide substitution model. The scale bar indicates 0.1 substitutions per site. **b** *Goniastrea aspera* (R): **b1** A small isolated colony, which appears brown due to the zooxanthellae which it contains. Often this species occurs as larger colonies covering several meters in shallow water habitats that may sometimes be quite turbid. **b2** Closeup of polyps showing cream-coloured lobes of the oral disc. **b3** Closeup of the skeleton showing the complex skeletal structure underlying each polyp. **c** *Fungia fungites* (R): **c1** The colony consists of a single polyp which is usually withdrawn during the day and expanded at night. **c2** Closeup of the mouth area, covered by sometimes multicoloured living tissue. **c3** The skeleton consists of closely spaced septa which support the living tissue. **d** *Galaxea fascicularis* (C): **d1** A large encrusting colony. **d2** Closeup of the polyps, which can be of diverse colours. *Galaxea* is unusual amongst corals in that the polyps are often extended during the day. **d3** The *Galaxea* skeleton differs considerably from those of many other massive corals in that only thin layers of coenostium link the individual polyps. **e** Portion of a colony of *Acropora millepora* (C). **f** Colony of *Acropora digitifera* (C). **g** Colony of *Porites lutea* (C*)*. **h** The sea anemone, *Aiptasia pallida*. **i** The starlet sea anemone, *Nematostella vectensis*. Photo credits are given in the Acknowledgements

We report the genome sequences of the Scleractinian corals *Goniastrea aspera* (R) (Fig. 1b1–b3), *Fungia sp*. (R) (Fig. 1c1–c3), and *Galaxea fascicularis* (C) (Fig. 1d1–d3) using a whole genome shotgun strategy based on libraries with insert sizes in the 250-bp to 15-kb range (Additional file 2: Table S2). The estimated genome sizes, according to k-mer analyses, displayed ~2×-fold variation amongst coral species. The observed SNP rates within a genome fluctuated between 0.89 and 1.27%, which are high but broadly in line with values for other cnidarians for which genome sequence data are available. The assembled genomes comprised 764 Mb, 606 Mb, and 334 Mb, representing approximately 89%, 87%, and 62% of the estimated genome sizes for *Goniastrea* (R), *Fungia* (R), and *Galaxea* (C) respectively. In each case, the GC content was approximately 39% (Table 1; Additional file 2: Table S3); thus, base composition is remarkably consistent across the nuclear genomes of both robust and complex Scleractinian corals, whereas the base composition of the mitochondrial genome differs markedly between the two groups (Additional file 2: Table S4; Additional file 3: Figure S2) and more variation in base composition is seen in sea anemone nuclear genomes (Additional file 2: Table S3). De novo annotation of repetitive sequences revealed that transposable elements (TEs, ~ 98% of total repeats) are by far the dominant repeat types in cnidarian genomes, and as has been observed in many other lineages, larger genomes harbour higher proportions of transposons (Table 1; Additional file 2: Table S5). Therefore, TE expansion may underlie the observation that robust coral genomes (two in the present study, plus *Pachyseris speciosa* (Bongaerts et al., unpublished), *Favia favus (*Ying et al., unpublished), and *Stylophora pistillata* [20] at ~ 1 Gb, 900 Mb, and 457 Mb respectively) are generally larger than those of complex corals.

**Table 1:** Genome assembly and annotation statistics for the three sequenced coral genomes

		*Galaxea*	*Fungia*	*Goniastrea*
Assembled genome size (Mb)^a^		334	606	764
SNP rate		1.27	1.19	0.89
Scaffolds	Number	11,269	7424	5396
	N50 (kb)	87	323	518
	Largest (kb)	874	1804	2896
GC%		39.43	38.41	39.29
Number of genes^b^		22,418	38,209	35,901
Repeats^c^	Total repeat (%)	31.71	37.36	44.77
	Interspersed repeat (%)	30.41	35.59	42.91

^a^See Additional file 3: Table S3 for more detail

^b^See Additional file 3: Table S6 for more detail

^c^See Additional file 3: Table S5 for more detail

For each genome, annotation of protein-coding genes was accomplished by ab initio prediction, supported by ultra-deep transcriptome sequencing (~ 200 million reads per sample, Additional file 2: Table S2) and homologue-based analyses. In total, 35,901, 38,209, and 22,418 genes were identified from *Goniastrea* (R), *Fungia sp*.(R), and *Galaxea* (C) respectively (Table 1; Additional file 2: Table S6). Of these, 70% to 80% appeared to be complete and over 90% were found to have clear homologues from the NR database (Additional file 2: Table S7). The completeness of genome assemblies and gene models was assessed using the Core Eukaryotic Genes Mapping Approach (CEGMA) [26] and Benchmarking Universal Single-copy Orthologs (BUSCO) [27]. These assessments indicate that the core gene set in the three genomes from this study is within the same range as previously published cnidarian genomes (Additional file 2: Table S8). Moreover, biological names could be assigned to > 60% of genes from UniProt-Swissprot annotations (Additional file 2: Table S9). Well-defined PFAM-A protein domains were identified in approximately 65% of the annotated genes (Additional file 2: Table S10), which is within the same range as in a number of model organisms [28]. In total, unambiguous KEGG K numbers could be assigned to ~ 50% of genes (Additional file 2: Table S11), enabling comprehensive metabolic pathway analyses. The overall consistency in level of functional annotations indicates a consistent high quality of gene models that are suitable for gene content analyses. However, the variability in number of ab initio annotated genes likely reflects the general uncertainties associated with short-read-based assemblies, where gene number estimates can be biased by assembly and annotation artefacts [29], complicating direct comparisons of gene copy numbers amongst species. To provide broader perspectives on likely differences between complex and robust corals, the *Galaxea* (C) data were supplemented with genome data from three other members of the Complexa – *Acropora digitifera* [17], *Acropora millepora* (Ying et al., unpublished), and *Porites lutea* (Robbins et al., unpublished).

### Gene-based phylogeny and synteny across the Hexacorallia

Phylogenetic analyses of high-quality single-copy orthologous genes, making use of a recently developed general nucleotide substitution model (see “Methods”) [30], produced a tree (Fig. 1a) congruent with a monophyletic clade of complex and robust corals. Whilst it is widely recognised that maximum likelihood (ML) methods based on nucleotide substitution models most accurately represent the true underlying evolutionary processes [30] and are therefore superior to amino acid substitution models [31], they are usually not used for deeply diverged species due to concerns over sequence divergence saturation [32]. However, amino acid models have been demonstrated to be non-Markovian [31, 33], and their congruence with the underlying Markovian process operating on nucleotides is likely to be rare [34, 35]. This necessitates use of nucleotide-based models of sequence evolution. In order to apply nucleotide-based ML analyses to the coral dataset, 687 (of a total of 2573 identified by OrthoFinder) one-to-one orthologs matching the same SwissProt gene were selected using the criterion of > 60% target coverage. For the analyses, the general nucleotide model, which removes the unrealistic ubiquitous assumptions of stationarity and time-reversible conditions, was employed and time-heterogeneity permitted throughout the phylogeny. To avoid overfitting and reduce the computational burden, a progressive approach (including a model selection strategy) was adopted, starting with four taxa and gradually increasing the number to ultimately resolve the phylogeny relationships for species of interest. The model was fitted to each individual gene, and only genes that satisfied the identifiability conditions (see Methods) were retained, allowing robust inferences to be drawn. Ultimately, this resulted in 91 genes being used for reliable branch length estimation for the full phylogeny (Fig. 1a). The resulting consensus phylogenetic tree clearly separates robust corals (*Goniastrea* and *Fungia*) from complex corals (*Galaxea*, *Porites*, and *Acropora*) using the sea anemone *Nematostella* as outgroup. The same topology was obtained using IQ-TREE built-in amino acid models under partition mode (Additional file 3: Figure S4) [35, 36]. However, the modelling processes are not directly comparable between nucleotide and amino acid models, and the former should be strongly preferred in any future phylogenetic analyses that include more coral species.

**Fig. 2:**
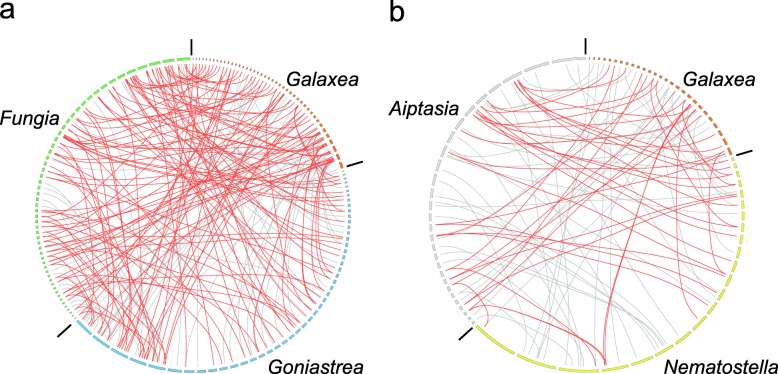
The significant preservation of gene collinearity between complex and robust corals. Synteny relationships are shown in the form of circle plots. In each plot, three species were chosen: two from the same lineage and another from a different lineage. The broken lines at the periphery of each circle represent scaffolds, with the length of each segment indicating the relative length of that scaffold. The top five most synteny block-rich scaffolds were selected from each species and were linked with the corresponding syntenic regions in the other species. Grey lines link syntenic blocks that were identified only in one pair of species, whilst red lines highlight syntenic blocks that were shared (minimum two overlapping syntenic orthologs) by two pairs of species. The number of syntenic blocks between robust (e.g. *Fungia*) and complex (e.g. *Galaxea*) corals (**a**) greatly exceeds that between the sea anemones *Aiptasia* and *Nematostella* (**b**)

To enable identification of conserved gene linkages in deep phylogenetic comparisons, synteny analyses were performed based on orthologous gene collinearity. Using the same microsynteny threshold as previously described [19, 37], 5045 (22.5%) *Galaxea* (C) genes were paired with genes in *Acropora digitifera* (C) in 642 syntenic blocks. Amongst these, 2891 (57.3%) *Galaxea* (C) genes were also identified as orthologs within syntenies to *Fungia* (R). The comparison between *Galaxea* (C) and *Fungia* (R) identified 4521 syntenic orthologs in 620 syntenic blocks. Counter-intuitively, levels of conservation between various pairs of coral species were relatively uniform (Fig. 2a; Additional file 2: Table S12), in that not only were large numbers of syntenic orthologous genes identified within lineage comparisons (e.g. Complexa versus Complexa), but comparably large numbers of such genes in cross lineage comparisons (Complexa versus Robusta). Considering the likely divergence times of the range of species studied (the complex/robust split has been estimated at ~ 415 MYA [3]), the extent of gene order conservation observed within the Scleractinia is remarkable. By contrast, a much lower level of conservation was detected between *Aiptasia* and *Nematostella* (Fig. 2b; Additional file 2: Table S12). Only 922 *Aiptasia* genes were found as syntenic genes to *Nematostella* in 153 syntenic blocks, amongst which 263 (28.5%) genes were shared with *Acropora digitifera* (C). Synteny analysis between *Aiptasia* and *Acropora digitifera* (C) placed 1376 orthologous genes in 224 syntenic blocks; note that the estimate from *Aiptasia* is consistent with recent published analyses [19].

Whilst the variable quality of the assembled genomes being compared complicates synteny analyses (fragmented genomes potentially reducing the apparent degree of synteny detected), this issue did not affect the major conclusions being drawn in the present case. Amongst the species included in the analyses, the *Galaxea* (C) and *Nematostella* genome assemblies are represented by the largest numbers of scaffolds, but the N50 for the *Nematostella* assembly was much longer than in the case of the *Galaxea* (C) assembly (Additional file 2: Table S3). Therefore, the limited extent of synteny observed in the anemone lineage was not an artefact of assembly quality, and despite accurate estimates of divergence times not being available, the analysis presented here provides compelling evidence that extensive intra- and inter-chromosomal rearrangements have occurred in the sea anemone lineage.

In contrast to sea anemones, the extensive synteny observed between complex and robust corals, a divergence that also occurred in deep time, suggests that ancestral gene arrangements may be better preserved in coral genomes than in other anthozoans so far examined. One major caveat to this, however, is that no comparable data are yet available for members of the other major anthozoan sub-class, the Octocorallia. Hopefully, this deficiency will be addressed in the near future, providing broader perspectives on ancestral gene organisation in the Anthozoa.

### Clustered organisation of HOX-related genes

**Fig. 3:**
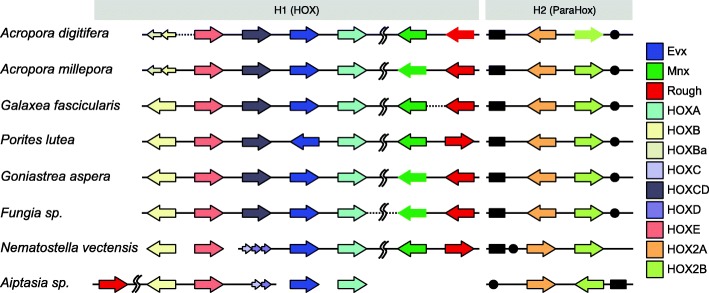
Organisation of HOX-related genes in corals and sea anemones. Relative positions and orientations of cluster H1 genes are largely conserved across taxa with the exception of *Aiptasia*. Arrows indicate the direction of transcription and colours distinguish between orthologous groups of genes identified by phylogenetic analysis. Genes that have been duplicated within a family are represented by multiple smaller arrows. The nomenclature used here for the H1 genes is based on Chourrout et al. [35] and Baumgarten et al. [20]; note that *Mnx1* is also known as *hlxB9*. Unconnected arrows represent different scaffolds (two from *Nematostella* and two from *Aiptasia*); dotted lines indicate genes that were assembled on a different scaffold from other H1 genes, but can be putatively placed in the cluster by comparing gene arrangement with other species; arrows without outline indicate a manually corrected gene model; vertical wavy lines represent a long genomic distance with 10–20 genes between. Gene order and orientation in ParaHox cluster H2 are highly conserved in corals, whilst local gene rearrangement is observed in both sea anemones. Black box and circle represent the *POMP* and *CD027* genes, respectively

Against the background of very limited overall synteny between corals and sea anemones, a cluster of HOX-related genes stands out as an apparent exception to this general pattern (Fig. 3). Homeobox gene clusters, each consisting of several HOX-related genes and (single) *Evx* and *Mnx1/HlxB9* genes, have previously been identified in both *Nematostella* [38, 39] and *Acropora* (C) [40–42]. Whilst the organisation of these genes is similar in *Nematostella* and *A. digitifera* (C), the distinct organisation of these genes in the *Aiptasia* genome [19] led to the suggestion that extensive genome rearrangements might be a common feature of anthozoan genomes and raised uncertainty concerning ancestral organisation of HOX-related genes. *Evx* is a member of the Antp superclass of homeobox genes (reviewed in [43]) that can be unambiguously identified based on a highly conserved homeobox sequence and is present as a single copy gene in every species included in the present study. Extensive examination of the genomic regions proximal to *Evx* orthologs led to the recognition of another (single copy) linked homeobox gene, *Rough* (Fig. 3), in several species. Vertebrate genomes do not encode a *Rough* gene, and the *Drosophila Rough* and *Mnx* (now known as *Extra extra* (*Exex*)) genes are unlinked. However, *Rough* and *Mnxa* are linked in the amphioxus genome [44, 45] and it has been suggested that this gene pair was part of a “super-HOX” cluster in the ancestral urbilaterian [45]. Identification of orthology relationships amongst the coral and sea anemone HOX-related genes by phylogenetic methods (Additional file 3: Figure S5; Additional file 4) allowed the overall organisation of these genes to be compared and revealed a general pattern shared by *Nematostella* and both complex and robust corals (Fig. 3; Additional file 2: Table S13). Synteny analyses indicate that the whole cluster is fully represented from complex corals to robust corals (*Goniastrea* confirmed, *Fungia* highly likely) and *Nematostella*. In the case of *Aiptasia*, the *Evx* gene is not linked to other HOX-related genes; it is located towards the centre of a scaffold that contains 157 predicted genes (data not shown). However, the identification of the *Rough* ortholog in this species enabled identification of the corresponding cluster of HOX-related genes for comparative analyses.

Whilst the synteny analyses presented here are consistent with quite different patterns of organisation of HOX-related genes in *Aiptasia* and *Nematostella*, our results imply that *Aiptasia* is atypical and that the cluster structure seen in corals and *Nematostella* reflects the ancestral state. In some ways, this is not particularly surprising as these two anemones are widely diverged in sea anemone phylogenies based on two nuclear and three mitochondrial genes [46]. Although some cases of inversion and duplication have clearly occurred, the apparent conservation of an anthozoan HOX-related cluster over at least 500 MY suggests that strong selection has acted to maintain the organisation of these genes, presumably reflecting conservation of function [42]. Note that the cluster of HOX-related genes in cnidarians is not orthologous with the “true” HOX cluster of bilaterians, as the cnidarian/bilaterian divergence predated the origins of the latter [39, 47].

In addition to the cluster of HOX-related genes discussed above (“H1” in Fig. 3), a second pair of HOX-related homeobox genes was identified in several corals as well as both sea anemones (although the orientation of the genes differs in the case of the sea anemones; “H2” in Fig. 3; Additional file 2: Table S13). The gene referred to here as *HOX2A* corresponds to *cnox2* in *A. millepora* [48], and this linkage was first identified in *Nematostella* [39, 47]. *HOX2A/cnox2* is the cnidarian homologue of the ParaHox gene *Gsx*, and *HOX2B* most closely matches *Xlox*– also a ParaHox gene—although it has been suggested that the cnidarian gene corresponds to both *Xlox* and the third ParaHox gene, *Cdx* [39]. This region is syntenic across all the species studied except for *Nematostella*; the genes flanking the ParaHox gene pair (*CD027* and *POMP*, which encode homologues of the Histone PARylation factor1 and the Proteosome maturation protein UMP1 respectively) are also conserved single-copy genes in each case (with the apparent exception of *Nematostella*, which has two copies of both *CD027* and *POMP*). The “H2” gene pair represents the cnidarian ParaHox cluster [49, 50], and the conservation of this genomic region across the range of species studied suggests that ParaHox diversification may have been incomplete at the time of the cnidarian/bilaterian divergence.

### Patterns of domain and gene distribution: expanded gene families are often tightly linked

**Fig. 4:**
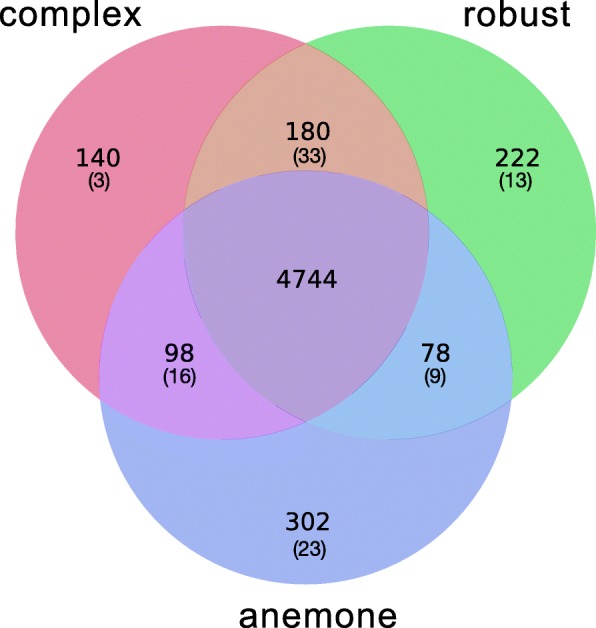
Venn diagram of shared and unique PFAM-A domains amongst sea anemones and complex and robust corals. Set membership counts shown without parentheses consider a domain to be present in a lineage if it is found in any of the species in that lineage. Numbers in parentheses indicate set memberships calculated with the requirement that the domain is present in all species in that group. In the situation where corals of the complex or robust lineages form a group with anemones, the domain is counted when it is present in all coral species and either of the sea anemones

To investigate whether complex corals, robust corals, and sea anemones differ substantially in terms of content of genes with defined functions, the distribution of PFAM-A protein domains (Fig. 4) amongst these three groups was examined. Whilst initial analyses identified hundreds of domains that appeared to be restricted to single lineages (e.g. 302 domains unique to anemones), more restrictive analyses requiring unique domains to be supported by all species within each of the three groups resulted in much smaller numbers, indicating that some domains have more limited distributions (Table 2; Additional file 2: Table S14). In total, 33 PFAM-A domains were found in every Scleractinian but not in either anemone. Amongst these, the OLF (olfactomedin-like) domain, which is typically involved in cell-cell interactions and cell adhesion, is particularly interesting in that 10–20 copies were present in corals, but this domain was absent from both sea anemones. Notably, a number of PFAM-A domains directly associated with histidine biosynthesis were restricted to robust corals—this topic is explored in greater depth below.

**Table 2:** Lineage restricted PFAM-A domains

	Domain	Description		Domain	Description
Present in ANEMONE not in coral	DUF3445	Protein of unknown function (DUF3445)	Present in COMPLEX not in robust corals	BCL_N*	BCL7, N-terminal conserver region
	DUF853	Bacterial protein of unknown function (DUF853)		SR-25*	Nuclear RNA-splicing-associated protein
	IF3_N	Translation initiation factor IF-3, N-terminal domain		Toxin_R_bind_N*	Clostridium neurotoxin, N-terminal receptor binding
	CHZ	Histone chaperone domain CHZ		DUF4557	Domain of unknown function (DUF4557)
	DUF455	Protein of unknown function (DUF455)		Nucleoplasmin*	Nucleoplasmin
	MotA_ExbB	MotA/TolQ/ExbB proton channel family		CBM_11*	Carbohydrate binding domain (family 11)
	COX7a	Cytochrome c oxidase subunit VIIa		Microtub_assoc*	Microtubule associated
	PNMA	PNMA		Drc1-Sld2*	DNA replication and checkpoint protein
	SLBB	SLBB domain		Prefoldin_3*	Prefoldin subunit
	5-nucleotidase	5′-nucleotidase		CutA1*	CutA1 divalent ion tolerance protein
	MacB_PCD	MacB-like periplasmic core domain		DUF2414*	Protein of unknown function (DUF2414)
	Glyco_trans_4_2	Glycosyl transferase 4-like		Dynein_IC2*	Cytoplasmic dynein 1 intermediate chain 2
	HtrL_YibB	Bacterial protein of unknown function (HtrL_YibB)		MIF4G_like*	MIF4G like
	Glyco_transf_17	Glycosyltransferase family 17		TAN*	Telomere-length maintenance and DNA damage repair
	DUF1762	Protein of unknown function (DUF1762)		DUF2201	VWA-like domain (DUF2201)
	FAM216B	FAM216B protein family		MGC-24*	Multi-glycosylated core protein 24 (MGC-24)
	Resistin	Resistin		Hint_2	Hint domain
	DUF3598	Domain of unknown function (DUF3598)		Mem_trans*	Membrane transport protein
	PDH	Prephenate dehydrogenase		DUF4606*	Domain of unknown function (DUF4606)
	YbjQ_1	Putative heavy-metal-binding			
	CBM_20	Starch binding domain			
	IF2_N	Translation initiation factor IF-2, N-terminal region			
	IlvN	Acetohydroxy acid isomeroreductase, catalytic domain			
Present in CORAL not in anemone	OLF	Olfactomedin-like domain	Present in ROBUST not in complex corals	RAG1	Recombination-activation protein 1 (RAG1)
	LRRNT	Leucine rich repeat N-terminal domain		DUF2341	Domain of unknown function (DUF2341)
	Myb_DNA-bind_3	Myb/SANT-like DNA-binding domain		Toxin_60	Putative toxin 60
	HTH_19	Helix-turn-helix domain		*HisG*	*ATP phosphoribosyltransferase*
	Parvo_coat_N	Parvovirus coat protein VP1		Glt_symporter*	Sodium/glutamate symporter
	ScpA_ScpB	ScpA/B protein		NTPase_I-T*	Protein of unknown function DUF84
	MFS_3	Transmembrane secretion effector		XG_Ftase*	Xyloglucan fucosyltransferase
	BBE	Berberine and berberine like		Phospholip_A2_2*	Phospholipase A2
	LBR_tudor	Lamin-B receptor of TUDOR domain		Polysacc_lyase	Polysaccharide lyase
	HATPase_c_4	ATP-dependent DNA helicase recG C-terminal		*PRA-CH*	*Phosphoribosyl-AMP cyclohydrolase*
	PSDC	Phophatidylserine decarboxylase		Sigma70_r4_2*	Sigma-70, region 4
	STAG	STAG domain		HTH_Tnp_Tc3_2*	Transposase
	GH3	GH3 auxin-responsive promoter		IGPD*	*Imidazoleglycerol-phosphate dehydratase*
	Glutaminase	Glutaminase		EURL	EURL protein
	Smoothelin	Smoothelin cytoskeleton protein		*Histidinol_dh*	*Histidinol dehydrogenase*
	DUF1982	Domain of unknown function (DUF1982)		Hexapep_2*	Hexapeptide repeat of succinyl-transferase
	MRP-L28	Mitochondrial ribosomal protein L28		DUF4094	Domain of unknown function (DUF4094)
	Tocopherol_cycl	Tocopherol cyclase		*HisG_C*	*HisG, C-terminal domain*
	Sec39	Secretory pathway protein Sec39		DUF1864	Domain of unknown function (DUF1864)
	DUF3496	Domain of unknown function (DUF3496)		Phage_T7_tail	Phage T7 tail fibre protein
	DUF4613	Domain of unknown function (DUF4613)		PRA-PH*	*Phosphoribosyl-ATP pyrophosphohydrolase*
	HK	Hydroxyethylthiazole kinase family		Lipase_GDSL_3	GDSL-like Lipase/Acylhydrolase family
	Macro_2	Macro-like domain			
	DUF72	Protein of unknown function DUF72			
	SRP9–21	Signal recognition particle 9 kDa protein (SRP9)			
	PMT_2	Dolichyl-phosphate-mannose-protein mannosyltransferase			
	STAT_int	STAT protein, protein interaction domain			
	CNPase	2′,3′-cyclic nucleotide 3′-phosphodiesterase (CNP or CNPase)			
	UPF0066	Uncharacterised protein family UPF0066			
	Tnp_zf-ribbon_2	DDE_Tnp_1-like zinc-ribbon			
	Eno-Rase_NADH_b	NAD(P)H binding domain of trans-2-enoyl-CoA reductase			
	CR6_interact	Growth arrest and DNA-damage-inducible proteins-interacting protein 1			
	BRCA-2_OB3	BRCA2, oligonucleotide/oligosaccharide-binding, domain 3			

*Domains present in *Nematostella* or *Aiptasia*. See Additional file 2: Table S14 for more detail

Domains shown in italics represent domains involved in the histidine biosynthesis pathway

Although relatively few PFAM-A domains met the restrictive criterion of being present in all members of one of the groups (i.e. sea anemones or corals; complex corals or robust corals) but being absent from all members of the other group(s), comparative analyses revealed that 161 and 62 domains differed significantly in copy number between corals and anemones, and between complex and robust corals, respectively (see “Methods”; Additional file 2: Table S15 and S16; Additional file 3: Figure S6 and S7). Some differences between corals and sea anemones in domain counts are likely to be associated with calcification in the former—for example, the EGF_CA (calcium-binding EGF domain) is greatly expanded in all corals—whereas other differences in domain or gene distributions may be associated with the symbiotic lifestyle. The fact that *Acropora* is particularly enriched with respect to glycosyl transferase domains (Glyco_trans_1_4, Glycos_trans_1) has previously been documented [51]; it is now clear that this is a general feature of corals. However, using the size of gene classes alone as a criterion of difference may also be inappropriate in some cases, as the depth of the coral/anemone divergence suggests that some similarities may be consequences of convergent evolution rather than conservation of function.

The small heatshock protein (HSP20) family provides examples of uneven expansions not only between the complex and robust coral suborders, but also between representatives of the suborders. For example, despite similar numbers of HSP90 and HSP70 loci being present in all of the coral species studied, numbers of *HSP20/α-crystallin* genes varied more than twofold (Additional file 2: Table S17); the *Porites* (C) and *Goniastrea* (R) genomes encode 17 and 18 HSP20s respectively, whereas numbers were much smaller in *A. digitifera* (C) (9), *A. millepora* (C) (6), *Fungia* (R) (7), and *Galaxea* (C)(7). The same variability appears to hold for sea anemones; *Nematostella* encodes 18 HSP20s, whereas only five genes were identified in the *Aiptasia* gene set. Branching patterns observed in phylogenetic analyses (Additional file 3: Figure S8; Additional file 5) are consistent with the HSP20 sequences having undergone independent expansions in the range of anthozoans studied, and in many cases, the HSP20 paralogs were tightly linked. For example, many (14 of 17) of the *Porites* (C) HSP20 sequences fell into two major clades in phylogenetic analyses. Eight genes comprising one of these clades were on a single scaffold (Sc0000065) (Additional file 3: Figure S8); likewise, nine sequences comprising the major clade of *Goniastrea* (R) HSP20 sequences were on Sc0000418. Tight linkage of loci was also observed in corals with smaller numbers of HSP20 genes. For example, four of the nine *A. digitifera* (C) HSP20 genes were on a single scaffold (Additional file 2: Table S17); interestingly, transcription of the assumed orthologs of each of these genes was strongly upregulated *in A. millepora* (C) under CO_2_ stress [52]. Whilst data are presently available for relatively few species, an intriguing correlation can be seen between stress tolerance and numbers of HSP20 loci across the range of species studied here; those coral species containing greater numbers of HSP20 loci are substantially more stress tolerant than those with smaller numbers. For example, increases in terms of both colony abundance and spatial coverage following bleaching have been documented for *Porites lutea* (C) [53], and *Goniastrea fascicularis* (R) is one of the most stress-tolerant of Indo-Pacific corals. The same pattern holds for the two sea anemones for which whole genome data are available; by contrast with *Aiptasia*, *Nematostella* is remarkably stress tolerant, coping with wide ranges of both salinity (8.96 to 51.54 PSU) and water temperature (− 1 °C to 28 °C) (summarised in [54]). The apparent correlation between numbers of HSP20 loci in anthozoan species and stress tolerance deserves further exploration.

Tight linkage of paralogs, as observed in the case of HSP20 loci, appears to be a general characteristic of coral genomes—for example, in the case of the secreted and membrane associated type of carbonic anhydrase (CA) whose expansion has been associated with calcification [55]. All of the nine sequences of this type present in *Porites* (C) are located in a region of approximately 150 kb in the genome (Additional file 3: Figure S9). Tight linkage has also been observed in the case of independently duplicated homeobox genes (*Nk2*, *Dmbx1* and *Msx*) in *A. millepora* (C) [56].

### Robust corals have a fungal-like histidine biosynthetic pathway that is absent from complex corals and sea anemones

**Fig. 5:**
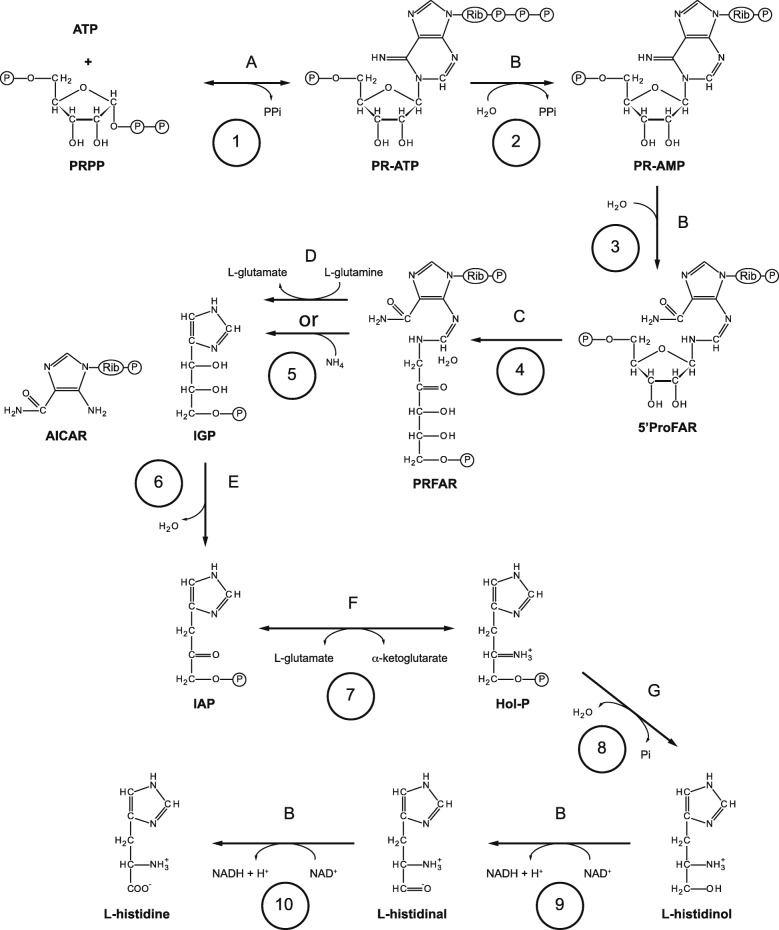
Histidine biosynthetic pathway in robust corals. The biochemical pathway by which histidine is synthesised from phosphoribosyl pyrophosphate is the same in plants, fungi, and bacteria, but some steps are brought about by unrelated proteins in different organisms. In robust corals, a fungal-like complement of enzymes is involved, the proteins responsible being (**a**) ATP phosphoribosyltransferase, (**b**) histidine biosynthesis trifunctional protein, (**c**) 5′ProFAR isomerase, (**d**) IGP synthase, (**e**) imidazoleglycerol-phosphate dehydratase, (**f**) histidinol-phosphate aminotransferase, and (**g**) histidinol-phosphate phosphatase. Abbreviations used: PRPP, phosphoribosyl pyrophosphate; ATP; adenosine triphosphate; PPi, pyrophosphate; PR-ATP, phosphoribosyl-ATP; PR-AMP, phosphoribosyl-AMP; 5′ProFAR, 1-(5-phosphoribosyl)-5-[(5-phosphoribosylamino) methylideneamino] imidazole-4 carboxamide; PRFAR, 5-[(5-phospho-1-deoxyribulos-1-ylamino)methylideneamino]-1-(5-phosphoribosyl) imidazole-4-carboxamide; IGP, imidazole-glycerol phosphate; AICAR, 1-(5′-phosphoribosyl)-5-amino-4-imidazolecarboxamide; IAP, imidazole-acetol phosphate; Hol-P, L-histidinol phosphate; Pi, phosphate; NAD+, oxidised nicotinamide adenine dinucleotide; NADH, reduced nicotinamide adenine dinucleotide

Amongst the eight species included in the comparative analyses, several (HisG, HisG_C, PRA-CH and Histidinol_dh) of the 22 PFAM-A domains detected only in robust corals were directly associated with histidine biosynthesis. Two other PFAM-A domains associated with the same pathway (PRA-PH and IGPD) were detected only in *Nematostella* and robust corals (Additional file 2: Table S18); the *Nematostella* hits we interpret as reflecting contamination on the basis of improbably high sequence identity with bacterial genes (for detail, see Additional file 2: Table S18 legend). Bacterial contamination of the *Nematostella* genome sequence is a known issue [57]; hence, six PFAM-A domains associated with the histidine biosynthetic pathway are restricted to robust corals. These six domains are associated with five (1, 2, 3, 6, and 9 in Fig. 5) of the ten steps involved in the histidine biosynthesis pathway that is common to bacteria, fungi, and plants but previously unknown in metazoans. Recovery of the gene predictions (Table 3; Additional file 2: Table S18) confirmed not only that the HisG and HisG_C domains are part of the same protein (ATP phosophoribosyl transferase; K00765), but also that another three of these domains (PRA-PH, PRA-CH, Histidinol_dh) reside in a single polypeptide; as in many fungi, both robust corals encode a multi-functional histidine biosynthesis protein (K14152) capable of catalysing steps 2, 3, 9, and almost certainly also step 10 of the pathway shown in Fig. 5. The IGPD PFAM-A domain is characteristic of imidazoleglycerol phosphate dehydratase (K01693; activity E, catalysing step 6 in Fig. 5). Searching SwissProt identified another enzyme associated with histidine biosynthesis present in robust corals; orthologs of histidinol phosphate aminotransferase (K00817; activity F, catalysing step 7 in Fig. 5) in *Goniastrea* (R) and *Fungia* (R). Completing the pathway, K04486 (= histidinol phosphatase; activity G and step 8 in Fig. 5) and K01663 (= glutamine amidotransferase/cyclase; activity) were identified in both *Goniastrea* (R) and *Fungia* (R) but were not restricted to robust corals in our analyses (Table 3; Additional file 2: Table S18). Thus, only one gene (K04486 = histidinol phosphatase; E3.1.3.15B) associated with histidine biosynthesis is present in a number of complex corals; and synteny is conserved in this region between robust and complex corals (Additional file 2: Table S19). The *Nematostella* genome also encodes a homologue of glutamine amidotransferase/cyclase (K01663), but there was no evidence to suggest its presence in any complex coral or *Aiptasia*. The numerous metabolic interconnections that exist lead us to suggest that these latter genes function primarily in nucleotide metabolism rather than histidine biosynthesis in complex corals and *Nematostella*. Thus, whereas the pathway is complete in the two robust corals studied, the available data imply that, as in the case of bilaterians, neither complex corals nor sea anemones are capable of de novo histidine biosynthesis.

**Table 3:** Genes involved in histidine biosynthesis pathway in cnidarians

Species	KEGG K Identifier	Gene ID	Activity (Fig. 5)	Step catalysed (Fig. 5)	SwissProt Accession ID	Match species	Gene description
*Fungia*	K00765	ffun1.m4.9038	A	1	Q75AK8	*Ashbya gossypii*	ATP phosphoribosyltransferase
	K14152	ffun1.m4.19036	B	2, 3, 9 and 10	P45353	*Komagataella pastoris*	Histidine biosynthesis trifunctional protein
	K01814	ffun1.m4.11161	C	4	Q6C2U0	*Yarrowia lipolytica*	1-(5-phosphoribosyl)-5-[(5-phosphoribosylamino)methylideneamino] imidazole-4-carboxamide isomerase
	K01663	ffun1.m4.971	D	5	Q9SZ30	*Arabidopsis thaliana*	Imidazole glycerol phosphate synthase hisHF
	K01693	ffun1.m4.26536	E	6	P28624	*Phytophthora parasitica*	Imidazoleglycerol-phosphate dehydratase
	K00817	ffun1.m4.6504	F	7	A5FFY0	*Flavobacterium johnsoniae*	Histidinol-phosphate aminotransferase
	K04486	ffun1.m4.13871	G	8	O14059	*Schizosaccharomyces pombe*	Probable histidinol-phosphatase
*Goniastrea*	K00765	gasp1.m3.565	A	1	Q99145	*Yarrowia lipolytica*	ATP phosphoribosyltransferase
	K14152	gasp1.m3.3160	B	2, 3, 9 and 10	P45353	*Komagataella pastoris*	Histidine biosynthesis trifunctional protein
	K01814	gasp1.m3.11564	C	4	Q10184	*Schizosaccharomyces pombe*	1-(5-phosphoribosyl)-5-[(5-phosphoribosylamino)methylideneamino] imidazole-4-carboxamide isomerase
	K01663	gasp1.m3.19737	D	5	Q9SZ30	*Arabidopsis thaliana*	Imidazole glycerol phosphate synthase hisHF
	K01693	gasp1.m3.16481	E	6	Q12578	*Candida glabrata*	Imidazoleglycerol-phosphate dehydratase
	K00817	gasp1.m3.19230	F	7	Q11VM5	*Cytophaga hutchinsonii*	Histidinol-phosphate aminotransferase
	K04486	gasp1.m3.11323	G	8	O14059	*Schizosaccharomyces pombe*	Probable histidinol-phosphatase
*A.millepora*	K04486	1.2.15090	G	8	O14059	*Schizosaccharomyces pombe*	Probable histidinol-phosphatase
*Galaxea*	K04486	gfas1.m1.2962					
*Porites*	K04486	plut2.m8.12019					
*Nematostella*	K01663	NEMVEDRAFT_ v1g235787	D	5	Q9SZ30	*Arabidopsis thaliana*	Imidazole glycerol phosphate synthase hisHF

See Additional file 2: Table S18 for more detail

**Fig. 6:**
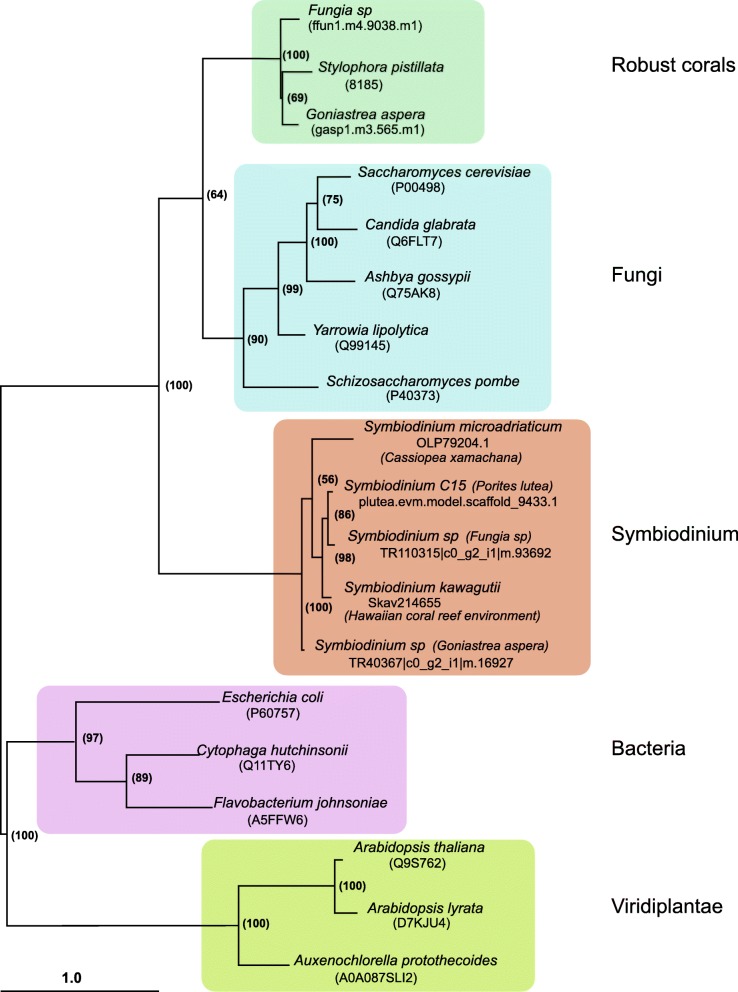
Phylogenetic analysis resolves the robust coral ATP phosphoribosyltransferases from their fungal and *Symbiodinium* homologues. ATP phosphoribosyltransferase proteins catalyse the first step in the histidine biosynthetic pathway, but the robust coral sequences are clearly resolved from those of representatives of other kingdoms of life in phylogenetic analyses. IQ-TREE was applied to generate the unrooted tree shown and automatic model selection chose LG + G4 as the best model. Numbers on nodes represent UFboot values based on 1000 iterations. Branch lengths indicate the expected number of amino acid substitutions per site. It is clear that robust coral proteins form a tight clade closest to fungal proteins, whereas proteins derived from *Symbiodinium* strains that are endosymbiotic with robust corals were most similar to those from other *Symbiodinium* isolates. In the case of *Symbiodinium* strains, the host species is indicated in parentheses, the exception being *S. kawagutii*, which, although isolated in association with the coral *Montipora verrucosa* (C), is now thought to be non-symbiotic [127]

Whilst de novo histidine biosynthesis has not previously been described in any metazoan, this property is widespread in fungi, plants, and bacteria, which use essentially similar pathways with only minor variations (Fig. 5). Both robust corals studied here encode homologues of each of the seven gene products comprising the histidine biosynthetic pathway in the yeast *Saccharomyces cerevisiae* and many other fungi and, in most cases, the best SwissProt database matches of the coral gene products were with fungal sequences, similarity typically being 40–60% and coverage > 80% (Table 3; Additional file 2: Table S18). Whilst these similarities raise the possibility of contamination, the coral sequences differ substantially from their counterparts in *Symbiodinium* [58–61] (the most likely source of any contamination), fungi, or other organisms. For example, phylogenetic analyses (summarised in Fig. 6) of the ATP phosphoribosyltransferase data (Additional file 2: Table S20; Additional file 3: Figure S10; Additional file 6) from a range of organisms clearly resolve the robust coral sequences from those of fungi and *Symbiodinium* strains, as well as from plant and bacterial sequences. Hence, the similarity with homologous fungal sequences is likely to be a consequence of phylogeny (fungi are the closest relatives of metazoans in which histidine biosynthesis has been documented) rather than contamination.

**Fig. 7:**
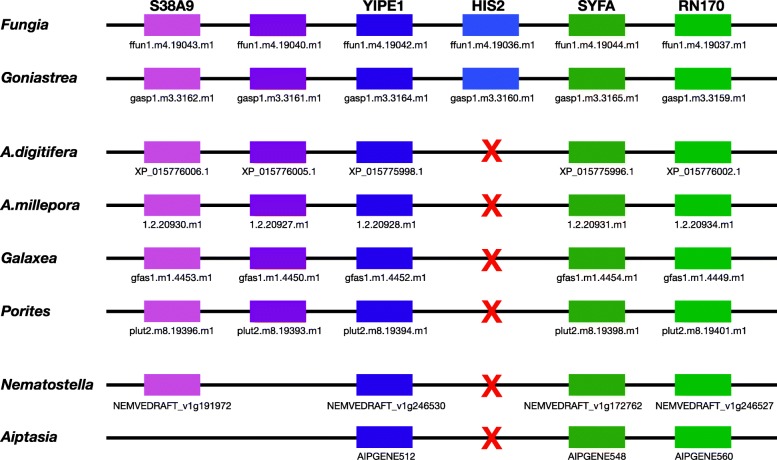
Loss of histidine biosynthesis trifunctional protein (HIS2, K14152) in complex corals and sea anemones. Syntenic regions surrounding the robust coral K14152 locus are conserved amongst robust corals, complex corals, and sea anemones, but K14152 has been lost in the latter two groups. Gene names identified from a blast search against the SwissProt database are shown at the top of the figure. Gene identifiers from each species are displayed beneath the coloured boxes. Relative positions of syntenic orthologs are aligned, and blank spaces represent missing syntenic orthologs. Red crosses are used to indicate that blast searches (using Evalue threshold 0.1) of those syntenic regions were conducted with both the robust coral K014152 nucleotide and protein sequences but did not detect any homology

The coral genes implicated in histidine biosynthesis are scattered throughout the genome (i.e. are on different scaffolds) rather than linked and, with the sole exception of that encoding imidazoleglycerol phosphate dehydratase (step 6 in Fig. 5; K01693), all contain introns (Additional file 2: Table S18). Note that the robust coral K01693 sequences were well resolved from bacterial, *Symbiodinium*, and other K01693 sequences in phylogenetic analyses (Additional file 2: Table S21; Additional file 3: Figure S11 and S12; Additional file 7), ruling out the possibility of contamination or lateral gene transfer. Moreover, examination of the genomic contexts of the robust coral histidine biosynthesis genes revealed synteny between robust and complex corals in the surrounding regions (Additional file 2: Table S19), consistent with gene loss having occurred in the latter (Fig. 7).

Searching the genome assemblies allowed the identification of syntenic blocks of genes surrounding five of the histidine biosynthesis genes in *Fungia* (R) and *Goniastrea* (R) (Additional file 2: Table S19), all but one of which (K04486) were not found in complex corals. The corresponding syntenic blocks of genes (but lacking the histidine biosynthesis gene) were identified in at least one complex coral (Additional file 2: Table S19), and some were also found in sea anemones. The genes neighbouring K00765, K01663, K01814, and K14152 in the robust corals can be matched as direct syntenic orthologs in complex coral genomes. K04486 is the only histidine pathway gene which is also found in complex corals, and in this case, synteny around the gene is shared between the robust and complex corals.

To verify that complex corals have actually lost the histidine pathway genes, sequence similarity searches were carried out on the regions between YIPE1 and SYFA (which flank the K14152 genes in robust corals; Fig. 7) in complex corals and sea anemones. These searches failed to detect any sequences homologous to K14152 (or any other gene) in that region in any complex coral or sea anemone, confirming that the loss(es) of K14152 in complex corals and sea anemones did not occur recently.

Although only two robust corals were included in the comparative survey described here, analyses of published and publicly available data support the hypothesis that histidine biosynthesis is a general property of robust corals and is likely to be an ancestral trait in the Scleractinia. Based on the genome annotation of Voolstra et al. [20], the complete pathway is also present in the robust coral *Stylophora pistillata* (Additional file 2: Table S22) [20]. Whilst it is not possible for us to directly demonstrate that the histidine biosynthesis pathway of robust corals is functional, a preliminary analysis of the publicly available transcriptome data for corals [9, 62, 63] provides further support for the hypothesis that a complete histidine pathway is present and functional in robust corals but not in complex corals or sea anemones (Additional file 2: Table S23). Moreover, the proteins that constitute the putative histidine biosynthetic pathway in *Fungia* and *Goniastrea* have all of the residues associated with function in the corresponding SwissProt reference sequences (Additional file 2: Table S24; Additional file 8). Draft genome assemblies for two representatives of the Corallimorpharia, sister order to the Scleractinia [10, 64], were recently reported [65], and although neither corallimorpharian genome encodes a complete histidine biosynthesis pathway, all of the necessary genes are present in one or other (or both) species (Additional file 2: Table S22), suggesting that the complete pathway is ancestral. Thus, a yeast-like histidine biosynthesis pathway is likely to be ancestral in the Scleractinia and ubiquitous across the Robusta—we hypothesise that it has been lost in complex corals and sea anemones rather than gained via lateral transfer. Histidine biosynthesis is an energetically demanding process, and there are numerous examples of genes being lost when their function is redundant, as utilisation of energy to produce non-functional proteins will presumably be selected against. Thus, it was advantageous for complex corals and sea anemones as well as “higher” animals (members of the Bilateria) to lose the pathway.

### Significance of these findings for coral research

The results presented here demonstrate how comparative genomics can inform understanding of biological characteristics of corals, including stress tolerance and host-symbiont interactions. Although so far data are only available for a limited number of species, the observation that anthozoans showing higher stress tolerances have larger numbers of HSP20 loci than do their more stress-sensitive counterparts is intriguing and worthy of further exploration. The fact that robust corals have a complete, and therefore presumably functional, histidine biosynthetic pathway means that they are not dependent on the resident photosymbiont (or heterotrophy) for supply of this “essential” amino acid, whereas this is the case with complex corals.

Because both robust and complex clades contain numerous symbiotic genera, only further research will clarify whether the pattern of presence/absence of the histidine biosynthesis pathway reported here is universal, and if it is, how it can be explained.

## Conclusions

The most significant implication of these comparative analyses is that, uniquely amongst animals, robust corals are capable of de novo histidine biosynthesis. Previously, the only known difference between corals with respect to biosynthetic capacity was the lack of the enzyme cystathionine β-synthase (suggesting a requirement for cysteine) in *Acropora* spp. (C) but not in other corals [17]. Whilst these metabolic differences may play roles in the selection of compatible *Symbiodinium* strains, experimental support for this idea is presently lacking. Indeed, the robust corals studied here host strains of clade C and clade D *Symbiodinium* (Additional file 1: Table S1), as do many complex corals, including *Acropora* and *Galaxea*. Note, however, that enormous variation exists within the clades (particularly clade C), and few genome data are available, so the possibility of metabolic influences on strain selection cannot be dismissed.

Based on comparative analyses of protein families that are represented in both *S. pistillata* (R) and *A. digitifera* (C), it has been suggested that many genes have been independently duplicated in the two corals [20]. However, the tandem organisation of several expanded gene families reported here suggests that concerted evolution might be at least partly responsible for the patterns observed when the corresponding sequences are subjected to phylogenetic analysis.

Both the amino acid and nucleotide-based analyses strongly support the separation of the robust and complex clades and the implied relationships amongst complex corals are consistent with recent phylogenetic studies [5, 66, 67]. The branch length leading to *A. digitifera* (C) (particularly evident in the nt-based tree) is surprising given that the fossil record implies a relatively recent origin of the genus (~ 55 MYA) [68, 69]. Nevertheless, the phylogenetic and synteny analyses are consistent with corals forming a tight grouping by comparison with sea anemones. However, sea anemones are an ancient and highly diverse lineage, clearly represented in the Cambrian fossil record [70, 71], within which *Nematostella* and *Aiptasia* are only distant relatives [72–74], so extensive divergence at the genome level should perhaps have been anticipated. Genome sequence data for a more representative range of sea anemones are required in order to determine whether extensive genome rearrangements are the norm, or whether *Aiptasia* and *Nematostella* are truly atypical in this respect.

## Methods

### Sample collection and sequencing

Single colonies of *Galaxea fascicularis*, *Goniastrea aspera*, and *Fungia sp*. were collected near Orpheus Island, Far North Queensland, Australia, during November 2012. They were subsequently maintained in an aquarium at the Orpheus Island Research Station of James Cook University for a few days until they spawned and (*Symbiodinium*-free) sperm could be collected. Genomic DNA was isolated at James Cook University using the phenol method in April–May 2014. Illumina paired-end and mate-pair libraries with insert sizes in the range of 250 bp to 15 kb were prepared according to the manufacturer’s protocol at the Australian Genome Research Facility (AGRF), Melbourne, Australia. Sequencing was performed on an Illumina HiSeq2500. Table S2 in Additional file 2 summarises the library types and sizes on which the assemblies were based. In total, 81.9 Gb (152× coverage), 171.6 Gb (245× coverage), and 190.6 Gb (222× coverage) of sequence data were generated for *Galaxea fascicularis*, *Fungia sp.*, and *Goniastrea aspera* respectively. To facilitate genome annotation, RNA samples from *Galaxea fascicularis*, *Fungia sp.*, and *Goniastrea sp.* were collected from adult coral tissues from Orpheus Island and processed and sequenced at AGRF, Melbourne.

### Genome assembly

FastQC [75] was applied for quality checking of every library. In addition, paired-end read quality, genome size, and genomic features were assessed using sga-preqc package [76]. Adaptors and low-quality bases were trimmed using libngs [77] with a minimum quality of 20 and a minimum read size of 130 bp. Only reads with sufficient quality from both pairs were retained. The genome assemblies were performed using ALLPATHS-LG [78] v52188 in haplodify mode. Gapcloser v1.12-r6 [79] was employed afterwards for additional scaffolding. Randomly selected de novo assembled transcripts were mapped to these de novo assemblies, as a result of which many were identified as duplicated copies (data not shown). This suggested that both haplotypes were present in part of the assembly despite the effort of the assembler to haplodify the sequences. Therefore, we used Haplomerger [80] to merge the two parental alleles into a single reference sequence. Finally, a blast approach was conducted to remove small redundant scaffolds less than 1 kb in length.

### Mitochondrial genome identification

Mitochondrial genome sequences for 17 robust corals and 39 complex corals were obtained from NCBI nucleotide database (Additional file 2: Table S4). To identify assembled mitochondrial scaffolds, coral genome sequences used in the present study were blasted against mitochondrial sequences from a close relative (Additional file 2: Table S4).

### Transcriptome assembly

Raw RNA-seq reads were trimmed by the same methods as DNA reads. Trinity c2.0.6 [81] was then applied for de novo assembly (TDN) and genome-guided assembly (TGG). Default parameters were used except for jaccard_clip and strand-specific library type options. Similar TDN transcripts were merged using cd-hit [82, 83] with 90% identity threshold. Because RNA samples from adult tissues are a mixture of coral and *Symbiodinium* RNA molecules, we applied PSyTrans [84], which is based on support vector machine classification, to separate host (coral) and symbiont (*Symbiodinium*) transcripts from TDN transcripts. The GC content for the whole transcriptome before and after separation is shown in Additional file 3: Figure S1.

### Genome annotation

The gene models were generated by ab initio prediction based on carefully selected training genes and external evidence.

Firstly, PASA [85] was applied to assemble TDN and TGG transcripts to the genome, followed by transdecoder [86] to produce a set of likely ORFs. Only complete ORFs containing both plausible start (ATG) and stop codons were selected. This resulted in 18,723 complete ORFs for *Galaxea*. For *Fungia* and *Goniastrea*, since RNA samples were collected from closely related species, this step produced many fewer complete ORFs adequate for subsequent analyses. To overcome this problem, we chose to run MAKER2 [87] using TDN and TGG transcripts as transcript evidence and proteins from the uniref90 database [88] for protein alignment. This yielded 32,208 and 39,568 complete ORFs for *Fungia* and *Goniastrea* respectively.

Secondly, these complete ORFs were blasted against the SwissProt database using E-value threshold 1E−20. We retained full-length complementary DNAs (fl-cDNA) whose target coverages and query coverages are greater than 80% and 70%, respectively. These fl-cDNAs were subjected to the following multiple filtering steps: (i) multiple exon transcripts coding for peptides containing at least 100 amino acids were required and transcripts overlapping the same genomic loci were removed; (ii) redundant fl-cDNAs were merged using cdhit with 80% similarity threshold on translated proteins, and the longer fl-cDNAs were retained; (iii) putative transposable elements were excluded based on transposonPSI [89] and hhblits [90] searches to transposon databases; (iv) we employed the perl script prepare_golden_genes_for_predictors.pl from JAMg [91] to enhance the accuracy of PASA predictions which made use of a splice aware aligner (exonerate) and output refined gene models. We randomly selected ~ 1000 refined gene models as a training dataset, and the rest were set aside for testing purposes.

Finally, the MAKER2 [87] annotation pipeline was run for ab initio prediction. The training gene set was used to train AUGUSTUS [92] and SNAP [93]. The resulting parameters were employed by corresponding programs from MAKER. The combined TDN and TGG transcripts were provided as EST evidence, and the proteins downloaded from uniref90 database were taken as external evidence for protein alignment. Finally, putative transposons in the gene model were removed as described above.

Repetitive elements were detected from two analyses for all the genomes compared in this study. Firstly, a de novo repeat library was generated with Repeat-Modeller (Version 1.0.8) [94] with default parameters. This library was combined with RepBase databases [95] and used as input for RepeatMasker [96] to identify repeat categories and locations. A summary of repeat components is presented in Additional file 2: Table S5.

### Homologue search against public protein databases

Throughout the analyses, we performed similarity searches against three public protein databases using BLASTP with an E-value cut-off of 1E−05. The annotated coral proteins were used as query, and the curated database proteins were used as target. These databases are as follows: (1) The high-quality curated Universal Protein Resource (UniProt) SwissProt database [88] was our major resource to indicate gene functions and queried first. We defined the target (query) coverage as the percentage of the target (query) length in the alignment. Gene biological descriptions were assigned by their best E-value hit. (2) The UniProt TreMBL protein database [88] was queried for proteins that did not have significant hits from SwissProt. (3) The NCBI non-redundant protein database (NR) was downloaded from the NCBI ftp site [97]. The top 10 and 100 hits were retained from UniProt and NR database queries, respectively.

### Gene space completeness assessment

CEGMA software version 2.5 [26] was conducted to assess the completeness of genome assembly and annotated gene models. The download included the reference dataset of 248 ultra-conserved core eukaryotic genes (CEGs). The program was run with default parameters, which define the presence of a CEG in a query sequence if the outcome from the HMM search exceeds a pre-computed minimum alignment score, and the alignment covers over 70% of a CEG.

BUSCO software version 1.1 [27] was applied to further assess the completeness of genome assembly and annotated gene models. The program was run with default parameters and the eukaryotic gene set was chosen as reference dataset.

### Functional annotation

Functional annotation was performed by homologue searching of the protein domain PFAM-A database [98] and the Kyoto Encyclopedia of Genes and Genomes (KEGG) database [99]. HMMER (hmmer3) [100] was used to perform alignments to Pfam-A hmm profile, and protein domains with E-value and c-Evalue lower than 1E−05 were selected. KEGG K number (KEGG orthology KO identifier) assignment followed the algorithm described by Mao et al. [101], which selected the first UniProt (SwissProt, if not, TreMBL) hit that had a corresponding K number with E-value lower than 1E−05 and fewer than five lower E-value hits. ID mapping file, idmapping.dat.gz, was downloaded from UniProt ftp site [88]. An in-house-developed script (kindly provided by Francesco Rubino (f.rubino@uq.edu.au University of Queensland Australia) was used to convert UniProt hits to K numbers.

### Genome phylogeny construction

The first step in this process was the identification of orthologous groups (OGs) from the eight cnidarian species sampled in the present study using OrthoFinder (version 0.2.5) [102] with default parameters. Single-copy orthologous genes were identified from one-to-one relationship OGs, and the results filtered by requiring the same SwissProt gene name match with target coverage greater than 60%. Genes whose predicted protein sequence, when translated from the gene model GFF3 files, did not agree with the downloaded protein sequence were also excluded. This resulted in 687 high-quality single-copy ortholog groups.

The alignments to be used for phylogenetic analyses were prepared as follows. Protein sequence alignments were generated for the single-copy one-to-one orthologs described above using MAFFT v7 [103] with the E-INS-i strategy, MLOSUM62 matrix, and 1000 maxiterate. The corresponding protein coding sequence (CDS) alignments were derived from these protein alignments using functions implemented in PyCogent [104]. The former was used for amino acid (AA) model-based phylogeny construction, and the latter was used for nucleotide (NT) model based phylogeny construction.

Amino acid models of sequence substitution are conventionally employed for phylogenetic analyses of highly diverged lineages to reduce the potential impact of saturation of substitutions—the point past which any additional changes in sequence cannot be identified. In general, substitution models with a small number of character states will saturate earlier than models with larger numbers of states. For this reason, AA models typically have been preferred over NT models [32]. Unfortunately, it has been shown that biological sequence evolution violates fundamental assumptions of AA substitution models [31]. As a consequence, the validity of inferences made using AA models alone is suspect and a substitution model that operates on the DNA sequence is required. Accordingly, we employed both a conventional AA model-based approach and separately one using a nucleotide substitution model [30]. These are described in more detail below.

The continuous-time general Markov nucleotide (GN) substitution model [30] was employed for the NT-based phylogenetic analysis. This is a non-reversible and non-stationary model, properties that have been demonstrated to improve robustness of phylogenetic inference [30, 33]. Using GN allows drawing on mathematical results concerning model identifiability [30, 105] to establish that sufficient phylogenetic signal exists (i.e. that the sequences are not saturated) for robust inferences to be drawn. These conditions are of Diagonal Largest in Column [DLC, 105] and the existence of a unique mapping between continuous and discrete time Markov processes [30].

Important drawbacks in using GN arise from its large number of parameters. First, the computational time required for model fitting is considerably greater than that for standard models. Second, when a time-heterogenous substitution model is desirable, there is also a risk of over fitting [106]. The set of possible models ranged from a globally time-homogeneous model (a single rate matrix) to the maximally time-heterogenous model (a separate rate matrix per branch). For a single tree with 5, 6, or 7 taxa, the total number of possible models is 877, 21,147, and 678,570 respectively. To eliminate the issue of over fitting, we employed a model selection approach that uses the corrected Aikake Information Criteria (AICc) to identify the optimal model from the complete solution space. Because of computational limitations, we were only able to assess the complete solution space for a tree with five taxa, i.e. the optimal model was chosen from the 877 possible models.

The species phylogenetic tree for eight taxa was constructed using maximum-likelihood estimation based on GN [30] as implemented in PyCogent [104]. All possible tree topologies were evaluated for the five taxon cases (15 possible trees) outlined below. Each CDS alignment was split into three separate alignments, one for each codon position. For a given phylogenetic tree, a separate optimal model (described above) was identified for each codon position alignment and the log-likelihood for the tree for the CDS was the sum of the log-likelihoods from the three optimal models for the codon position alignments. (Only alignments that passed the model identifiability tests for all codon positions for all tree topologies were used.) The tree with the maximum-likelihood was chosen as the “best” tree for each CDS alignment and the likelihood weights method [107] was employed to determine the consensus tree and quantify support for different branching orders.

A sequential approach was adopted to resolve the branching order of the coral species. Firstly, a four taxa phylogenetic tree was generated for *Nematostella*, *Galaxea* (C), *A. digitifera* (C), and *Fungia* (R). *Nematostella* was used as an outgroup to indicate the root position for corals. Secondly, separate five taxa analyses were conducted to infer the position of *Goniastrea* (R) and *Porites* (C), relative to the four species in the tree. The results clearly placed *Goniastrea* (R) with the robust coral *Fungia*, and *Porites* (C) with complex corals (Additional file 3: Figure S3). This outcome allowed us to combine the two topologies unambiguously. Finally, *Aiptasia* was added to the sea anemone group with *Nematostella* and *A. millepora* (C) was clustered with *A. digitifera* (C), to complete the eight taxon phylogenetic tree, from which the branch length from GN was estimated based on the method described in Kaehler et al. [30].

In addition, a conventional AA substitution model-based phylogenetic analysis was undertaken by maximum likelihood using IQ-TREE 1.5.5 [35]. The protein alignments from one-to-one orthologs were concatenated into a supermatrix and partitioned by genes. The best partitioning scheme and evolutionary model for each partition was assessed by ModelFinder [108]. By default, ModelFinder chooses the model that minimises the Bayesian information criterion (BIC) score. To assess branch support, the ultrafast bootstrap approximation (UFboot) was used, with 1000 replicates [109].

### Synteny analyses

Conserved syntenic blocks between species were identified using the MCScanX [110] package based on collinearity of orthologous genes. The first step was blastall through blastp to identify homologous genes. The second step made use of gene location information to generate syntenic blocks containing a minimum of three collinear orthologous genes separated by no more than 10 non-orthologous genes. Circos v0.68 [111] was employed to draw syntenic blocks amongst selected species.

### HOX gene cluster analyses

We identified *HOX* genes as homeobox containing genes that matched best to a *HOX* gene in the SwissProt database. Putative *HOX* cluster genes were defined as consecutive *HOX* genes, of which two clusters were discovered in most of the species in the present study. Examining neighbouring upstream and downstream linked genes, we defined the *HOX* cluster H1 as multiple *HOX* genes linked with *Evx*, *Mnx*, and *Rough* genes, and the other cluster as H2. *HOX* gene sequences from Baumgarten et al. [19] were used as a reference to classify *HOX* genes. All HOX protein sequences were aligned with MAFFT. The resulting alignment was trimmed to a single 56 amino acid alignable region and used for maximum likelihood phylogenetic analyses using IQ-TREE. Tree visualisation was performed using the R package, ggtree [112].

A few *HOX* and *HOX*-related genes were missing from the gene model in some species. They were manually corrected using Blast-based methods, as follows. (1) The lengths of the protein coding regions annotated as *Rough* sequences from *A. millepora* and *Fungia* were approximately the combined size of Mnx1 and Rough proteins in other species. We confirmed that they were erroneous mergers and accordingly should be separated into *Mnx1* and *Rough* genes. (2) The *Rough* gene is not present in the *A. digitifera* gene model(s). We used the *A. millepora Rough* gene coding sequence as reference and identified one *Rough* exon at the corresponding location (99% nucleic acid identity with the *A. millepora* sequence). The genomic position where the second *Rough* exon is expected is located in a sequencing gap in the *A. digitifera* genomic scaffold. This suggests that the problem is due to incomplete assembly and that a *Rough* gene is present and linked to *Mnx1* in *A. digitifera*. (3) The *Mnx1* gene is not annotated in *Goniastrea*. From the HOX cluster H1 gene arrangement, we isolated the genome sequences at the expected *Mnx1* location and identified a *Mnx1* gene using the *Fungia Mnx1* sequence as reference. (4) The *A. digitifera HOX2A* gene (XP_015763498.1) was identified using the *A. millepora* sequence as reference. The first exon and 54 nt of the second exon of the *HOX2B* gene are also present on the same scaffold as the *HOX2A* gene in *A. digitifera*; the rest of the predicted sequence is missing due to the presence of a sequencing gap in the scaffold. Thus, *A. digitifera* is likely to have linked *HOX2A* and *HOX2B* genes, as is the case in *A. millepora.*

### Protein family analyses

Fisher-exact tests were carried out to find expanded PFAM-A domains in a lineage [37]. Tests were performed between coral (robust and complex corals) and anemones (*Nematostella* and *Aiptasia*); and between complex and robust corals. In each test, the background and specific domain content were calculated as the total number of genes that were identified as PFAM-A domain-containing and genes with a specific domain in the corresponding lineage respectively. The resulting two-sided *p* values were subjected to Benjamini-Hochberg multiple test correction (FDR) [113] and a FDR threshold of 0.01 was chosen for significantly enriched domains.

### Gene phylogenetic tree construction

For the phylogenetic analyses of other genes presented in the present study, the protein sequences were aligned using MAFFT, poorly aligned regions (< 20% alignable sequences) were trimmed, and the phylogeny was then constructed using IQ-TREE. ModelFinder was applied to find the best fit model and 1000 UFBoot replicates were used to generate node support values.

### Conserved domain and functional residue search

To identify functional residues, we searched the Conserved Domain Database (CDD) using the NCBI Batch Web CD-Search Tool [114, 115]. The query sequences included putative histidine biosynthesis proteins from *Fungia*, *Goniastrea*, and their best matching SwissProt proteins. In cases where the matching protein was from a fungal species, the homologous protein from the model organism *Saccharomyces cerevisiae* was used, on the basis that structure/function relationships have been most extensively studied in this species. The CD search outputs enabled identification of domains shared between reference and coral proteins, and the resulting alignments were manually inspected for the presence of functional residues in the robust coral proteins. In addition, two proteins (Additional file 2: Table S24 (b)) that have functional residue information in the UniProt database [88] were aligned and compared to their corresponding robust coral proteins (Additional file 8).

## Additional files

Additional file 1: **Table S1.** Summary of biological data for *Galaxea fascicularis, Fungia fungites* and *Goniastrea aspera*. Data from Veron (1986, 2000) unless otherwise noted. (DOCX 510 kb)

Additional file 2: **Table S2.** Sequencing libraries used for genome assembly and annotation. **Table S3.** Overview of genome assembly statistics for cnidarian genomes. Table S4 Mitochodrial genome identification and GC content. **Table S5.** De novo annotated repeat content data for various cnidarian genomes. **Table S6.** Comparison of gene model statistics amongst cnidarians. **Table S7.** Numbers of genes giving significant matches with proteins in the NR database. **Table S8.** Percentages of Core Eukaryotic Genes (CEGs) identified with CEGMA and BUSCO analyses for genome assembly and predicted transcripts. **Table S9.** Summary of SwissProt annotation data. **Table S10.** Summary of PFAM-A domain annotation data. **Table S11.** Distribution of genes assigned with KEGG K numbers. **Table S12.** Numbers of syntenic orthologous genes identified from selected species pairs. **Table S13.** Gene identifiers of HOX-related genes, other homeobox genes and linked genes. **Table S14.** Unique PFAM-A domains identified from coral versus anemone and complex versus robust coral comparisons. **Table S15.** Significantly expanded PFAM-A domains from coral versus anemone comparison. **Table S16.** Significantly expanded PFAM-A domains from complex versus robust coral comparison. **Table S17.** HSP20 domain containing genes in cnidarians. **Table S18.** Full list of genes involved in histidine biosynthesis identified in all species included in this study. **Table S19.** Conserved syntenic orthologs for histidine biosynthesis genes and their neighbouring genes amongst the eight cnidarians. **Table S20.** Sources of ATP phosphoribosyltransferase (K00765) data used for phylogeny construction. **Table S21.** Sources of Imidazoleglycerol-phosphate dehydratase (K01693, intronless) data used for phylogeny construction. **Table S22.** Histidine biosynthesis genes identified in additional anthozoan species. **Table S23.** Histidine biosynthesis genes identified in additional transcriptome data. **Table S24.** Identification of functional residues in putative histidine biosynthesis proteins from robust corals. (XLSX 120 kb)

Additional file 3: **Figure S1.** GC content of de novo assembled transcripts before and after PSyTrans treatment. **Figure S2.** GC content of mitochondrial genomes. **Figure S3.** Consensus phylogenetic trees estimated from GN model with node support. **Figure S4.** Comparison of consensus phylogenetic trees for the eight cnidarian species which are the focus of comparative analyses, estimated from the GN (A) and AA (B) models. **Figure S5.** Phylogenetic relationships amongst HOX-related genes in corals and anemones. **Figure S6.** Heatmap showing relative counts of genes that displayed significant differences between corals and sea anemones. **Figure S7.** Heatmap showing relative counts of genes that displayed significant differences between complex and robust corals. **Figure S8.** Phylogenetic relationships of HSP20 genes in corals and anemones (A) and arrangement of HSP20 genes in *Porites lutea* (B). **Figure S9.** Organisation of membrane associated carbonic anhydrase genes in *Porites lutea*. **Figure S10.** Alignment of ATP phosphoribosyltransferase (K00765) proteins. **Figure S11.** Phylogenetic tree of Imidazoleglycerol-phosphate dehydratase (K01693) proteins. **Figure S12.** Alignment of Imidazoleglycerol-phosphate dehydratase (K01693) protein sequences. (PDF 10350 kb)

Additional file 4: Hox protein sequences used for construction of the phylogeny shown in Additional file 3: Figure S5. (FASTA 11 kb)

Additional file 5: HSP20 protein sequences that were used for phylogeny construction shown in Additional file 3: Figure S8. (FASTA 21 kb)

Additional file 6: ATP phosphoribosyltransferase (K00765) protein sequences that were used for construction of the phylogeny shown in Fig. 6. (FASTA 6 kb)

Additional file 7: Imidazoleglycerol-phosphate dehydratase (K01693) protein sequences that were used for phylogeny construction shown in Additional file 3: Figure S11. (FASTA 5 kb)

Additional file 8: Amino acid sequences of robust coral proteins implicated in histidine biosynthesis and the corresponding SwissProt homologues that were used for the identification of functional residues (as shown in Additional file 2: Table S24). (FASTA 10 kb)
