# Supplementary material for: Comparative genomics reveals the distinct evolutionary trajectories of the robust and complex coral lineages
Source: Genome Biol. 2018 Nov 2;19:175. doi: 10.1186/s13059-018-1552-8 (PMC6214176; doi:10.1186/s13059-018-1552-8)
Supplement: Supplementary file 1 — Table S1. Summary of biological data for Galaxea fascicularis, Fungia fungites and Goniastrea aspera. Data from Veron (1986, 2000) unless otherwise noted. (DOCX 510 kb) [file 13059_2018_1552_MOESM1_ESM.docx]

**Additional file 1**

**Table S1:** Summary of biological data for *Galaxea fascicularis, Fungia fungites* and *Goniastrea aspera*. Data from Veron (1986, 2000) unless otherwise noted.

**References**

Babcock RC. Reproduction and distribution of two species of Goniastrea (Scleractinia) from the Great Barrier Reef Province. Coral Reefs. 1984;2:187-195.

Babcock RC, Bull GD, Harrison PL, Heyward AJ, Oliver JK, Wallace CC, et al. Synchronous spawnings of 105 scleractinian coral species on the Great Barrier Reef. Mar Biol. 1986;90:379-94.

[Brown BE](http://aquasymbio.fr/en/bibliography?f%5Bauthor%5D=1763), [Dunne RP](http://aquasymbio.fr/en/bibliography?f%5Bauthor%5D=1764), [Goodson MS](http://aquasymbio.fr/en/bibliography?f%5Bauthor%5D=1765), [Douglas AE](http://aquasymbio.fr/en/bibliography?f%5Bauthor%5D=1766). [Marine Ecology: Bleaching patterns in reef corals.](http://aquasymbio.fr/en/marine-ecology-bleaching-patterns-reef-corals) Nature 2000;404:142-143.

Huang H, Dong Z, Huang L, Yang J, Di B, Li Y, et al. Latitudinal variation in algal symbionts within the scleractinian coral Galaxea fascicularis in the South China Sea. Marine Biol Res. 2011;7:208-11.

Huang H, Dong ZJ, Huang LM, Zhang JB. Restriction fragment length polymorphism analysis of large subunit rDNA of symbiotic dinoflagellates from scleractinian corals in the Zhubi Coral Reef of the Nansha Islands. J Int Plant Biol. 2006;48:148-52.

Keshavmurthy S, Hsu CM, Kuo CY, Denis V, Leung JK, Fontana S, et al. Larval development of fertilized “pseudo-gynodioecious” eggs suggests a sexual pattern of gynodioecy in Galaxea fascicularis (Scleractinia: Euphyllidae). Zool. Stud. 2012;51:143-9.

Keshavmurthy S, Meng PJ, Wang JT, Kuo CY, Yang SY, Hsu CM, et al. Can resistant coral-Symbiodinium associations enable coral communities to survive climate change? A study of a site exposed to long-term hot water input. PeerJ. 2014;8:e327.

LaJeunesse TC, Bhagooli R, Hidaka M, DeVantier L, Done T, Schmidt GW, et al. Closely related Symbiodinium spp. differ in relative dominance in coral reef host communities across environmental, latitudinal and biogeographic gradients. Mar Ecol Prog Series. 2004;284:147-61.

LaJeunesse TC, Loh WKW, van Woesik R, Hoegh-Guldberg O, Schmidt GW, Fitt WK Low symbiont diversity in southern Great Barrier Reef corals, relative to those of the Caribbean. Limnol Oceanog 2003;48:2046-2054.

Lin Z, Chen M, Dong X, Zheng X, Huang H, Xu X, et al. Transcriptome profiling of Galaxea fascicularis and its endosymbiont Symbiodinium reveals chronic eutrophication tolerance pathways and metabolic mutualism between partners. Sci Reports. 2017; 7:42100.

Loya Y, Sakai K, Heyward A. Reproductive patterns of fungiid corals in Okinawa, Japan. Galaxea, Journal of Coral Reef Studies, 2009;11:119-129.

Sakai K. Gametogenesis, spawning, and planula brooding by the reef coral Goniastrea aspera (Scleractinia) in Okinawa, Japan. Mar Ecol Prog Series. 1997;22:67-72.

Silverstein RN, Correa AM, LaJeunesse TC, Baker AC. Novel algal symbiont (Symbiodinium spp.) diversity in reef corals of Western Australia. Mar Ecol Prog Series. 2011;422:63-75.

Tonk L, Sampayo EM, Weeks S, Magno-Canto M, Hoegh-Guldberg O. Host-specific interactions with environmental factors shape the distribution of Symbiodinium across the Great Barrier Reef. PLoS One. 2013;8:e68533.

Veron JEN. Corals of Australia and the Indo-Pacific. North Ryde, NSW, Australia, Angus and Robertson; 1986.

Veron JEN. Corals of the World. Townsville, QLD Australia, Australian Institute of Marine Science & CRR Qld Pty Ltd., 2000.

Ziegler M, Arif C, Burt JA, Dobretsov S, Roder C, LaJeunesse TC, et al. Biogeography and molecular diversity of coral symbionts in the genus Symbiodinium around the Arabian Peninsula. J Biogeog 2017;44:674-686.
