# Supplementary material for: Comparative genomics reveals the distinct evolutionary trajectories of the robust and complex coral lineages
Source: Genome Biol. 2018 Nov 2;19:175. doi: 10.1186/s13059-018-1552-8 (PMC6214176; doi:10.1186/s13059-018-1552-8)
Supplement: Supplementary file 3 — Figure S1. GC content of de novo assembled transcripts before and after PSyTrans treatment. Figure S2. GC content of mitochondrial genomes. Figure S3. Consensus phylogenetic trees estimated from GN model with node support. Figure S4. Comparison of consensus phylogenetic trees for the eight cnidarian species which are the focus of comparative analyses, estimated from the GN (A) and AA (B) models. Figure S5. Phylogenetic relationships amongst HOX-related genes in corals and anemones. Figure S6. Heatmap showing relative counts of genes that displayed significant differences between corals and sea anemones. Figure S7. Heatmap showing relative counts of genes that displayed significant differences between complex and robust corals. Figure S8. Phylogenetic relationships of HSP20 genes in corals and anemones (A) and arrangement of HSP20 genes in Porites lutea (B). Figure S9. Organisation of membrane associated carbonic anhydrase genes in Porites lutea. Figure S10. Alignment of ATP phosphoribosyltransferase (K00765) proteins. Figure S11. Phylogenetic tree of Imidazoleglycerol-phosphate dehydratase (K01693) proteins. Figure S12. Alignment of Imidazoleglycerol-phosphate dehydratase (K01693) protein sequences. (PDF 10350 kb) [file 13059_2018_1552_MOESM3_ESM.pdf]

Additional file 3

(a)

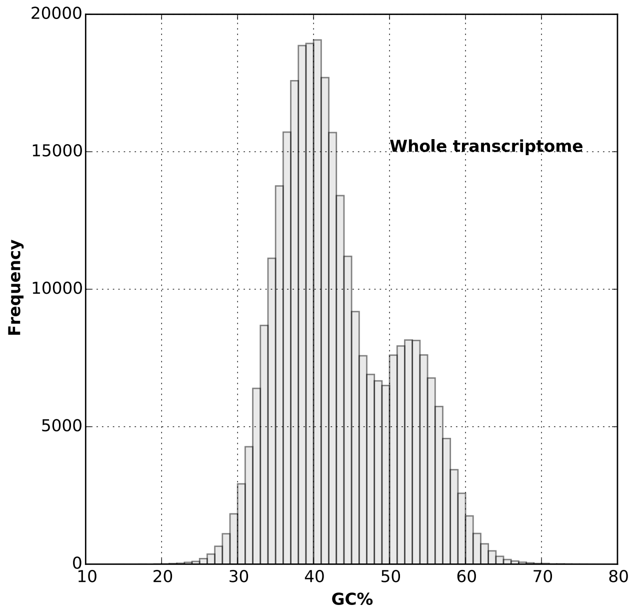

(b)

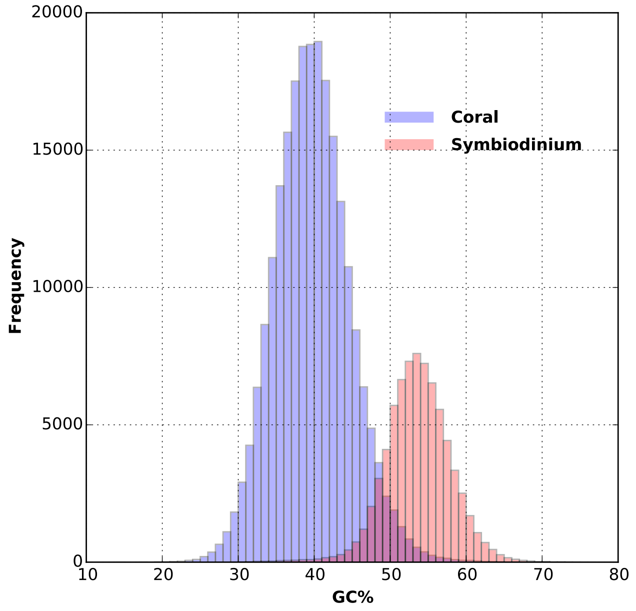

Figure S1: GC content of *de novo* assembled transcripts before and after PSyTrans treatment. (a) An apparent feature of Symbiodinium transcripts is that their GC content is higher than those from corals. Therefore, the GC content appears as a bimodal distribution from total RNA transcripts. (b) After PSyTrans separation, GC contents showed normal distributions from both coral and *Symbiodinium* transcripts.

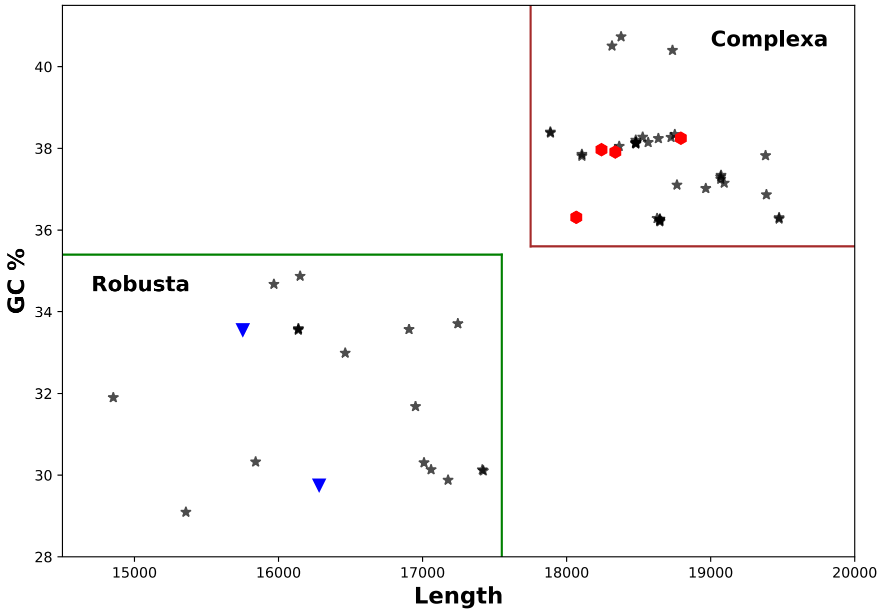

Figure S2: GC content of mitochondrial genomes. Black stars represent sequences obtained from the NCBI nucleotide database. Stars in the green box represent robust corals, and stars in the brown box represent complex corals. Blue triangles represent the two robust corals (*Fungia* and *Goniastrea*), and red pentagons represent the four complex corals (*A.digitifera, A.millepora, Galaxea* and *Porites)* used in the present study.

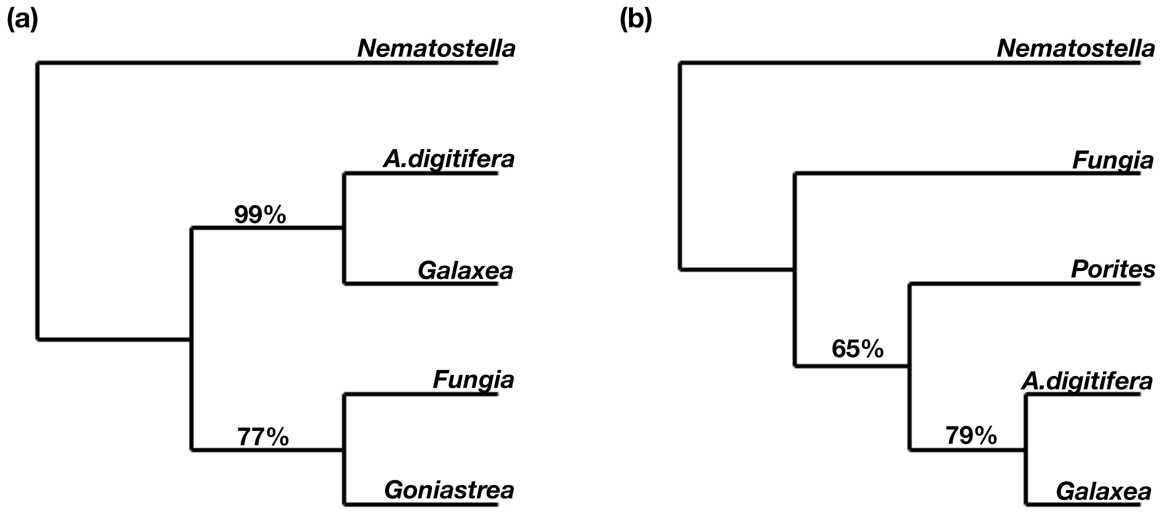

Figure S3: Consensus phylogenetic trees estimated from GN model with node support. The tree topology was firstly generated for *Nematostella, Galaxea, A.digitifera* and *Fungia*. *Nematostella* was used as an outgroup to indicate the root position for corals. The relative positions of *Goniastrea* (a) and *Porites* (b) to the four species were inferred separately. The percentages show node support based on the likelihood weights method described in Holland et al. 2006 [107].

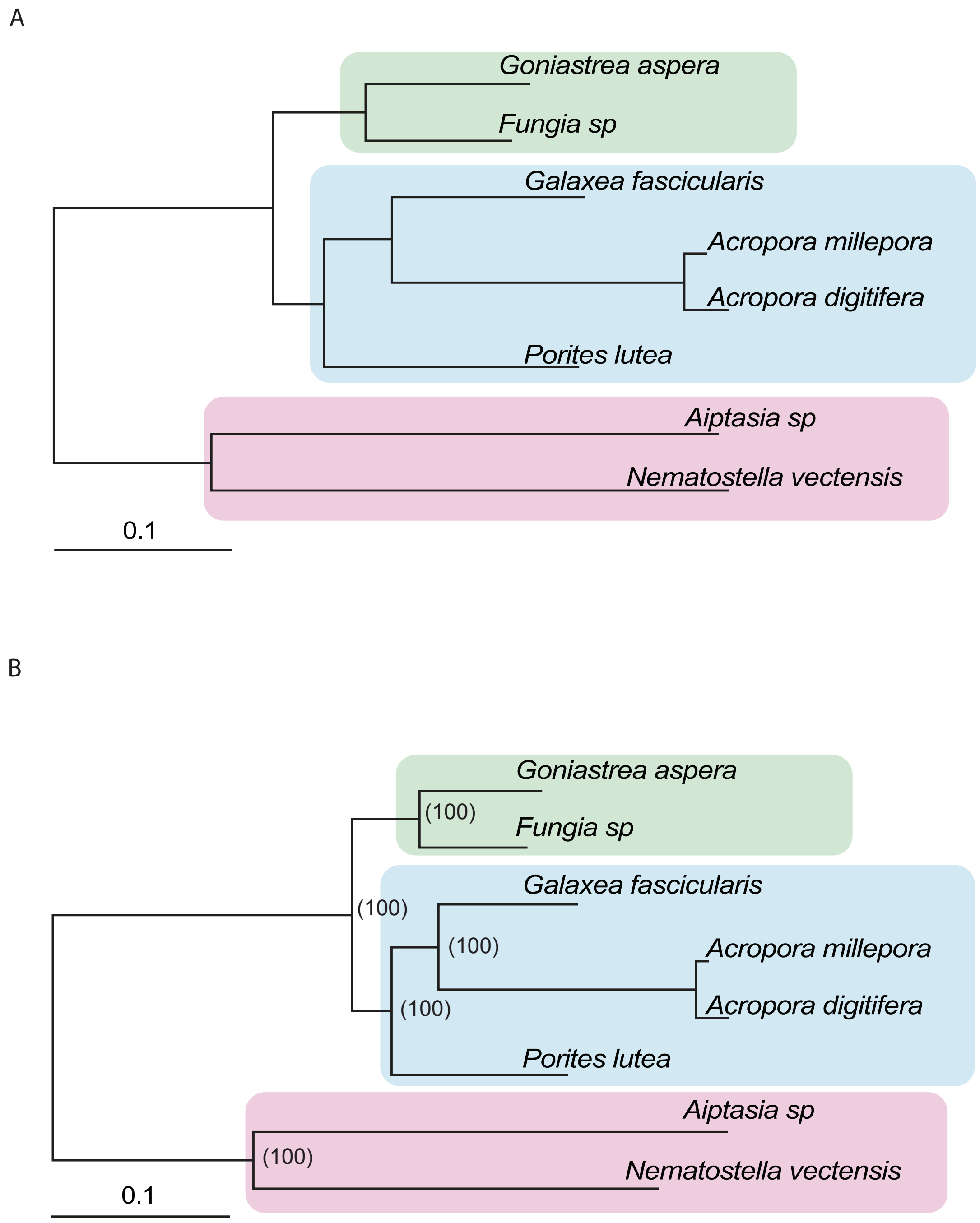

Figure S4: Comparison of consensus phylogenetic trees for the eight cnidarian species which are the focus of comparative analyses, estimated from the GN (A) and AA (B) models. Branch lengths of the GN-based tree were estimated from 91 protein coding sequence alignments, and node support values are shown in Figure S3. Protein alignments from the same 91 one-to-one orthologous genes were used to generate the AA-based tree. The partition model implemented in IQ-TREE was applied. Node support values (shown as percentages) were obtained from 1000 UFboot replicates.

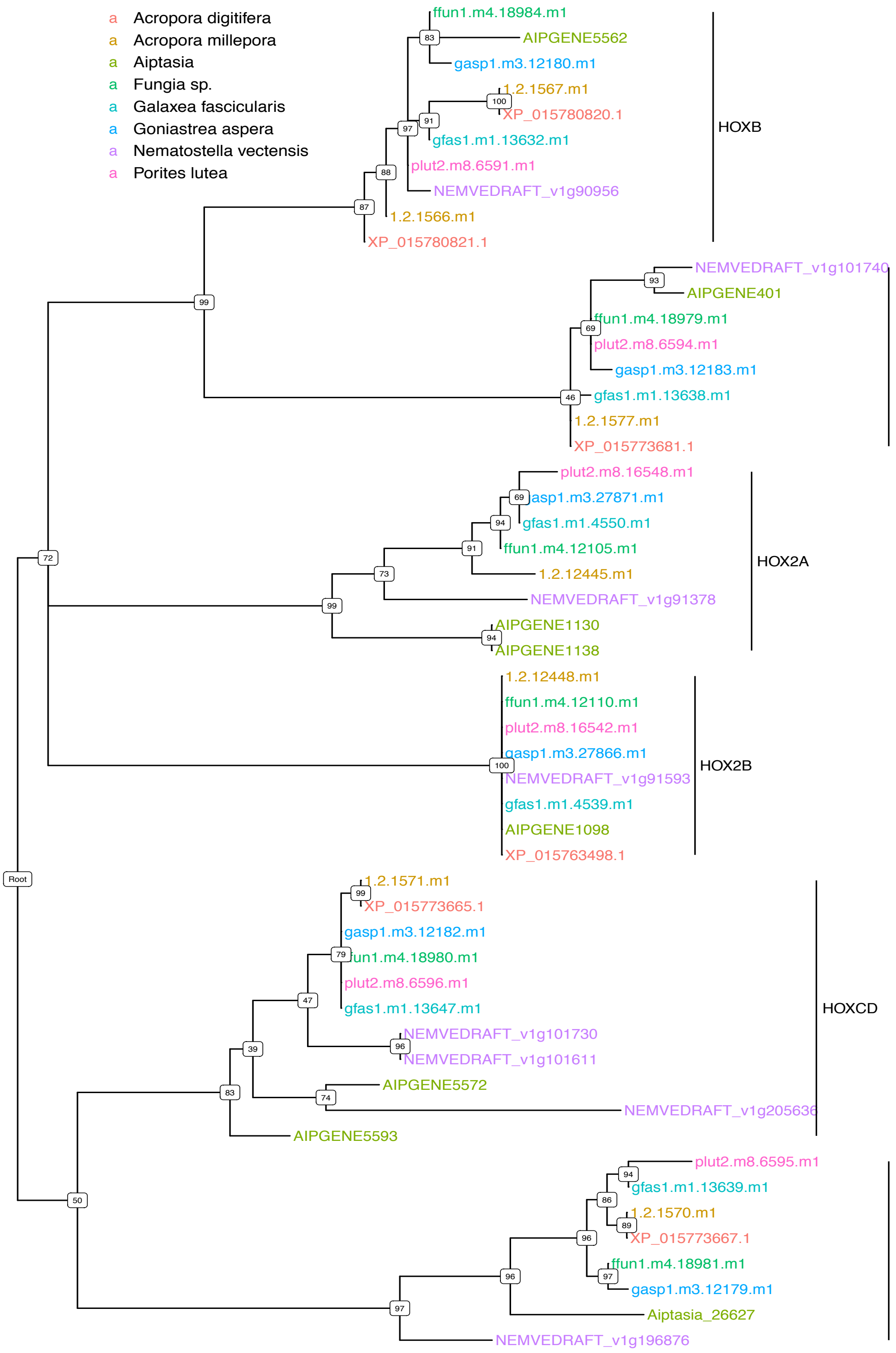

Figure S5: Phylogenetic relationships among HOX-related genes in corals and anemones. All genes are coloured according to species. Clades were identified and labelled according to the presence of sequences previously classified by Chourrout et al. 2006, Baumgarten et al. 2015 [19, 39]. Model (LG+G4) was selected as the best model. Node labels show support as a percentage of 1000 UFboot replicates. All node branch lengths less than 1E-5 were collapsed to polytomies. Sequences on which the tree was based are summarised in Additional file 2: Table S13 and provided in additional file 4.

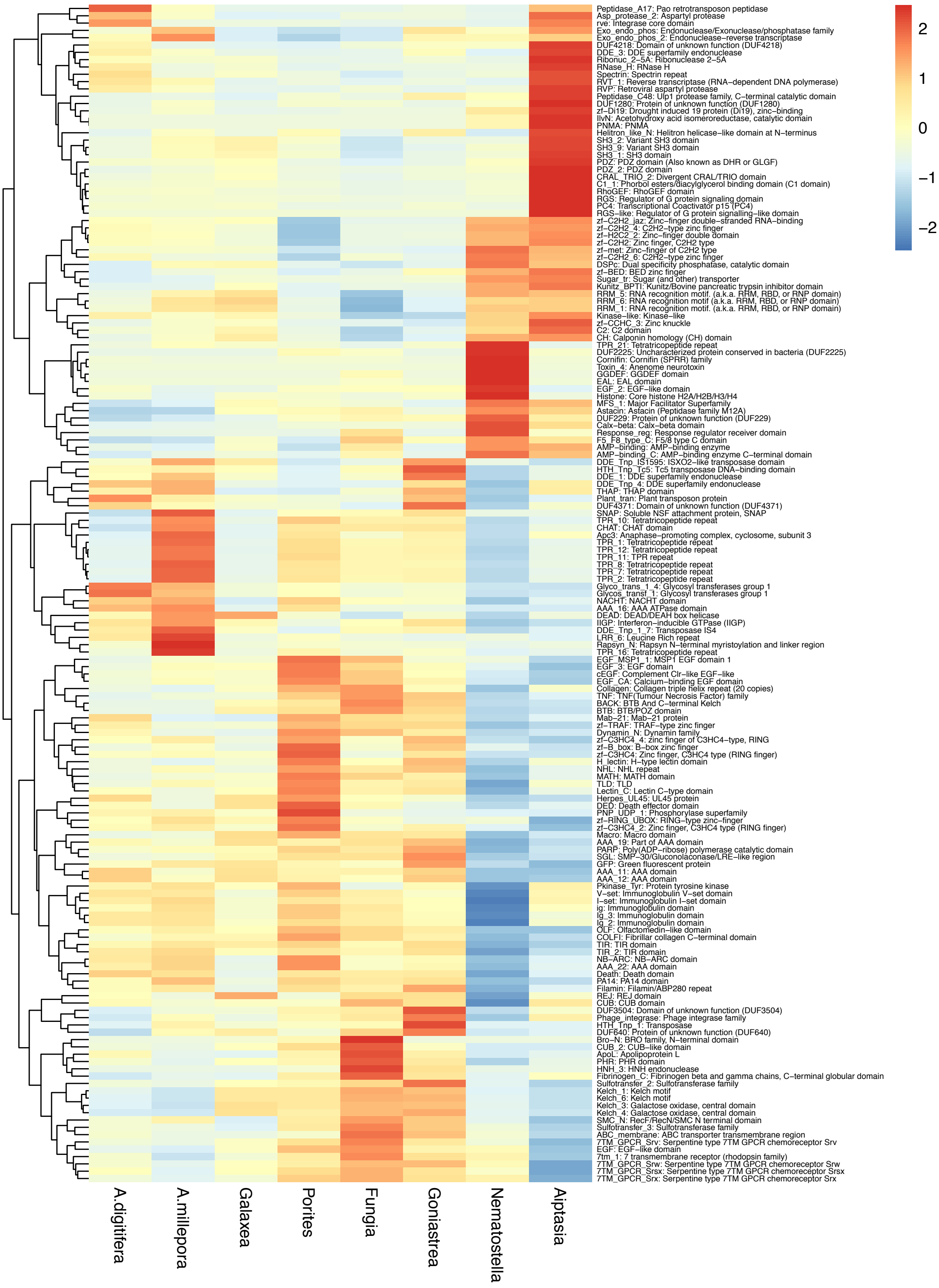

Figure S6: Heatmap showing relative counts of genes that displayed significant differences between corals and sea anemones. To obtain relative counts raw counts were centered and scaled for each row using the R function, scale. Included domains are those that differed significantly (FDR = 0.01 based on a Fisher's exact test) between anemones (*Nematostella* and *Aiptasia*) and corals (all other species). See Additional file 2: Table S15 for details of test statistics.

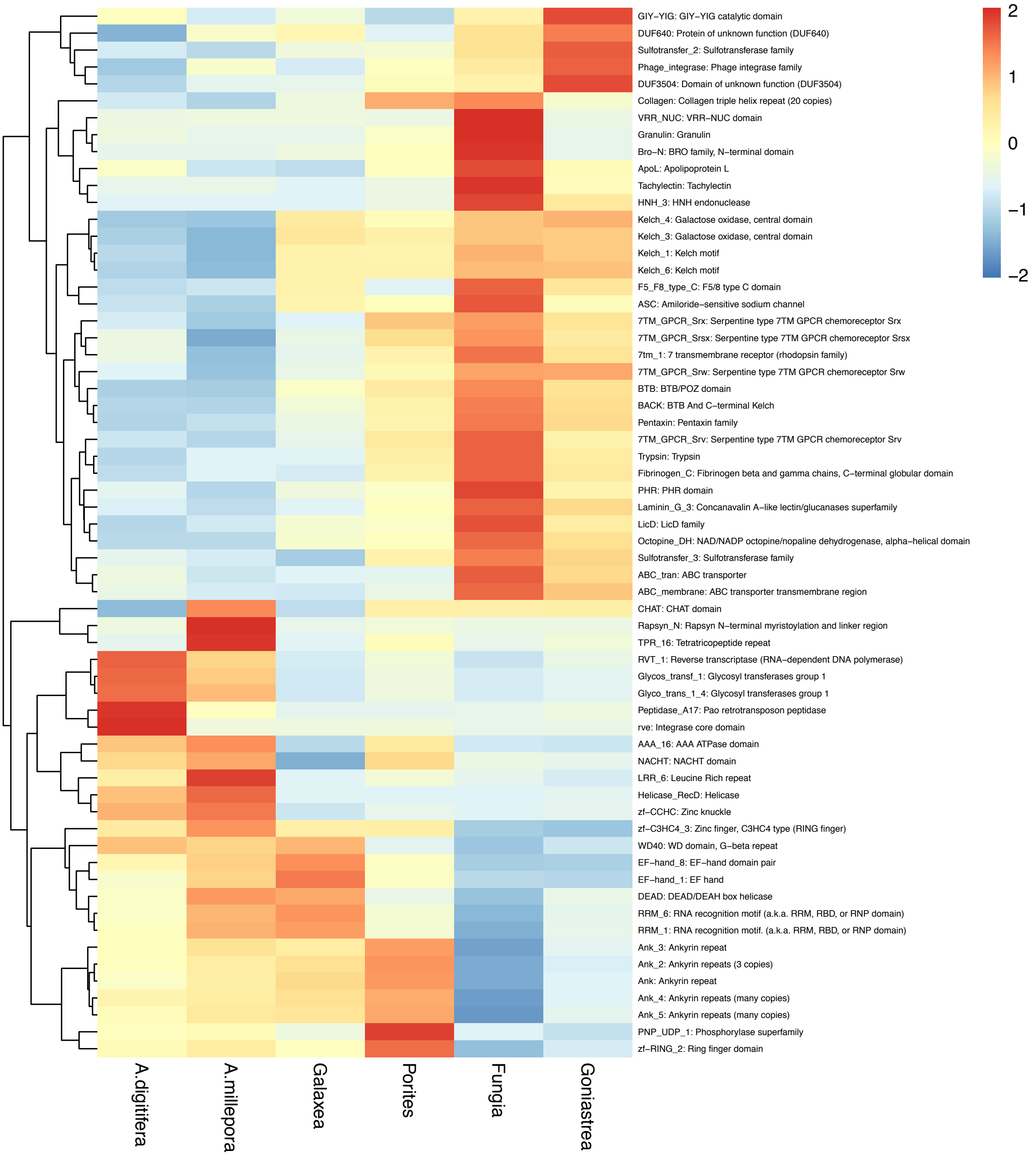

Figure S7: Heatmap showing relative counts of genes that displayed significant differences between complex and robust corals. To obtain relative counts raw counts were centered and scaled for each row using the R function, scale. Included domains are those that differed significantly (FDR = 0.01 based on a Fisher's exact test) between robust (*Fungia, Goniastrea*) and complex corals (*A.digitifera, A.millepora, Galaxea* and *Porites*). See Additional file 2: Table S16 for details of test statistics.

A

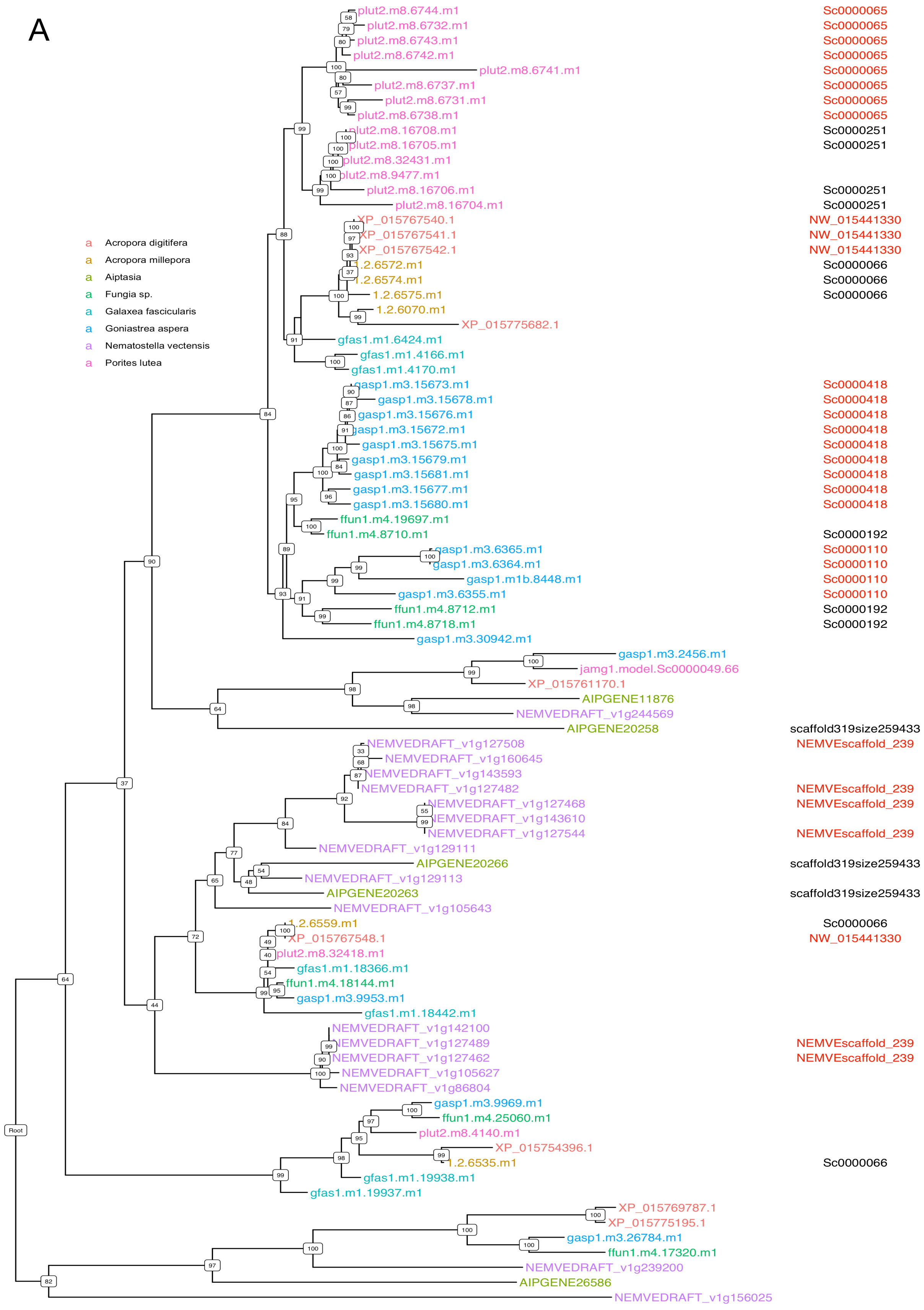

Acropora digitifera
Acropora millepora
Aiptasia
Fungia sp.
Galaxea fascicularis
Goniastrea aspera
Nematostella vectensis
Porites lutea
plut2.m8.6744.m1 Sc0000065
plut2.m8.6732.m1 Sc0000065
plut2.m8.6743.m1 Sc0000065
plut2.m8.6742.m1 Sc0000065
plut2.m8.6741.m1 Sc0000065
plut2.m8.6737.m1 Sc0000065
plut2.m8.6731.m1 Sc0000065
plut2.m8.6738.m1 Sc0000065
plut2.m8.16708.m1 Sc0000251
plut2.m8.16705.m1 Sc0000251
plut2.m8.32431.m1
plut2.m8.9477.m1
plut2.m8.16706.m1 Sc0000251
plut2.m8.16704.m1 Sc0000251
XP_015767540.1 NW_015441330
XP_015767541.1 NW_015441330
XP_015767542.1 NW_015441330
1.2.6572.m1 Sc0000066
1.2.6574.m1 Sc0000066
1.2.6575.m1 Sc0000066
1.2.6070.m1
XP_015775682.1
gfas1.m1.6424.m1
gfas1.m1.4166.m1
gfas1.m1.4170.m1
gasp1.m3.15673.m1 Sc0000418
gasp1.m3.15678.m1 Sc0000418
gasp1.m3.15676.m1 Sc0000418
gasp1.m3.15672.m1 Sc0000418
gasp1.m3.15675.m1 Sc0000418
gasp1.m3.15679.m1 Sc0000418
gasp1.m3.15681.m1 Sc0000418
gasp1.m3.15677.m1 Sc0000418
gasp1.m3.15680.m1 Sc0000418
ffun1.m4.19697.m1
ffun1.m4.8710.m1 Sc0000192
gasp1.m3.6365.m1 Sc0000110
gasp1.m3.6364.m1 Sc0000110
gasp1.m1b.8448.m1 Sc0000110
gasp1.m3.6355.m1 Sc0000110
ffun1.m4.8712.m1 Sc0000192
ffun1.m4.8718.m1 Sc0000192
gasp1.m3.30942.m1
gasp1.m3.2456.m1
jamg1.model.Sc0000049.66
XP_015761170.1
AIPGENE11876
NEMVEDRAFT_v1g244569
AIPGENE20258 scaffold319size259433
NEMVEDRAFT_v1g127508 NEMVEscaffold_239
NEMVEDRAFT_v1g160645
NEMVEDRAFT_v1g143593
NEMVEDRAFT_v1g127482 NEMVEscaffold_239
NEMVEDRAFT_v1g127468 NEMVEscaffold_239
NEMVEDRAFT_v1g143610
NEMVEDRAFT_v1g127544 NEMVEscaffold_239
NEMVEDRAFT_v1g129111
AIPGENE20266 scaffold319size259433
NEMVEDRAFT_v1g129113
AIPGENE20263 scaffold319size259433
NEMVEDRAFT_v1g105643
1.2.6559.m1 Sc0000066
XP_015767548.1 NW_015441330
plut2.m8.32418.m1
gfas1.m1.18366.m1
ffun1.m4.18144.m1
gasp1.m3.9953.m1
gfas1.m1.18442.m1
NEMVEDRAFT_v1g142100
NEMVEDRAFT_v1g127489 NEMVEscaffold_239
NEMVEDRAFT_v1g127462 NEMVEscaffold_239
NEMVEDRAFT_v1g105627
NEMVEDRAFT_v1g86804
gasp1.m3.9969.m1
ffun1.m4.25060.m1
plut2.m8.4140.m1
XP_015754396.1
1.2.6535.m1 Sc0000066
gfas1.m1.19938.m1
gfas1.m1.19937.m1
XP_015769787.1
XP_015775195.1
gasp1.m3.26784.m1
ffun1.m4.17320.m1
NEMVEDRAFT_v1g239200
AIPGENE26586
NEMVEDRAFT_v1g156025
Root

B

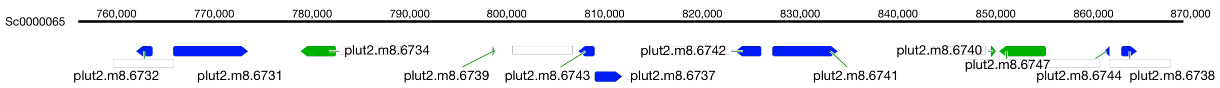

Figure S8: Phylogenetic relationships of HSP20 genes in corals and anemones (A) and arrangement of HSP20 genes in *Porites lutea* (B). (A) Genes are coloured according to species and corresponding scaffolds containing 2 or more HSP20 genes are shown in alternating colours. IQ-TREE was used to generate the maximum likelihood tree. Automatic model selection chose JTT+G4 as the best model and 1000 UFBoot replicates were used to generate support values shown as node labels. Sequences on which the tree was based are summarized in Additional file 2: Table S17 and provided in Additional file 5. (B) Close spacing of eight HSP20 paralogs is shown in blue with other genes shown in green.

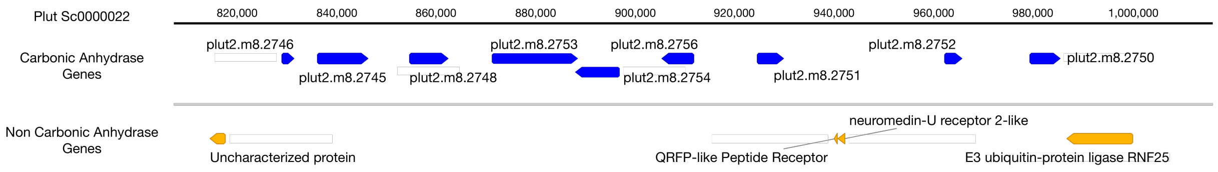

Figure S9: Organisation of membrane associated carbonic anhydrase genes in *Porites lutea*. Genes are shown as coloured blocks with the point designating the direction of transcription. Blue track shows the position and orientation of genes coding for carbonic anhydrases. The blue region spans ~160kb and contains just two non-carbonic anhydrase genes (shown in yellow).

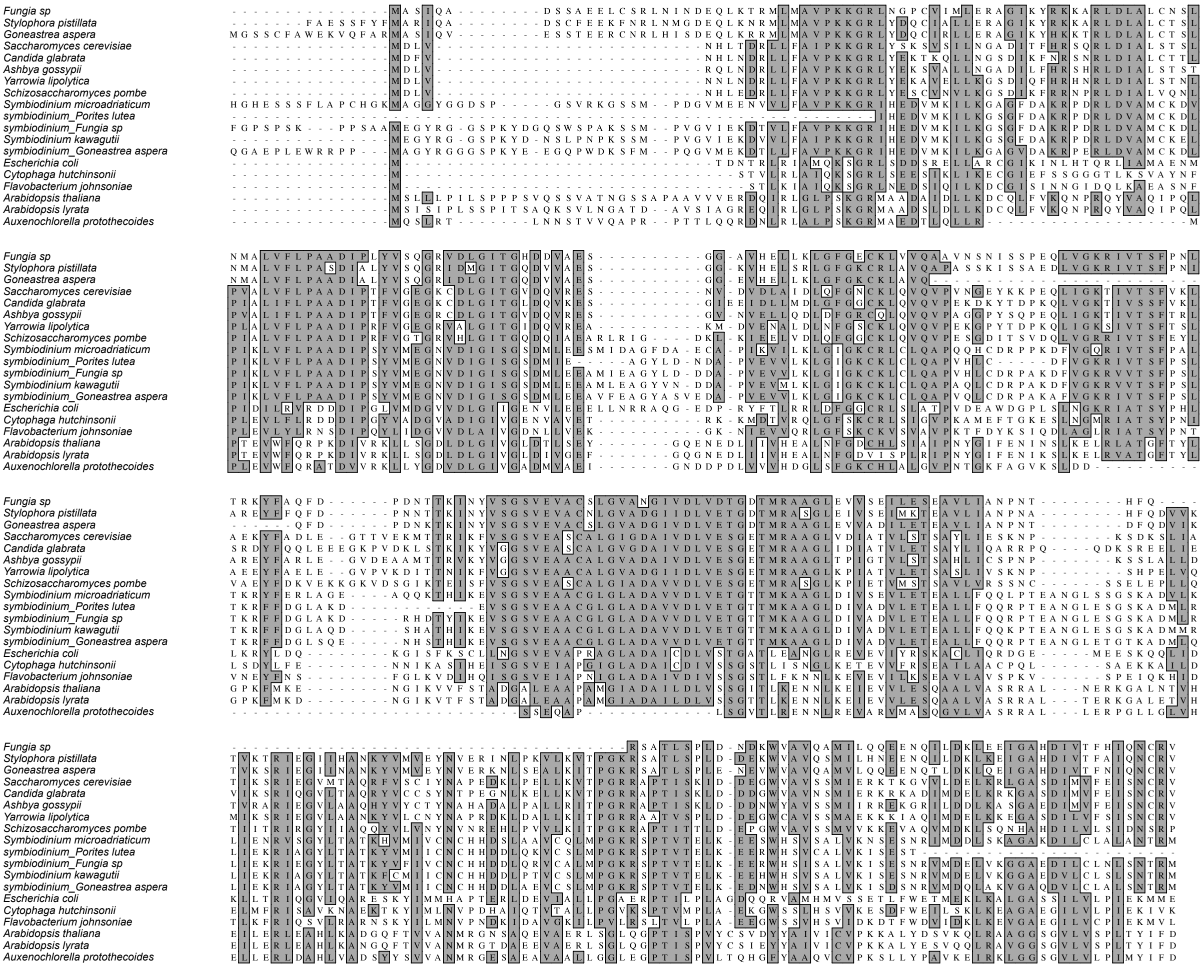

Figure S10: Alignment of ATP phosphoribosyltransferase (K00765) proteins. Poorly aligned columns with less than 20% alignable sequences were removed. The alignment shown was used to generate the phylogenetic tree Fig. 6. In the case of *Symbiodinium* strains, the host species are indicated after the underscore. Sequences are summarised in additional file 2: Table S20. Full length annotated protein sequences are provided in Additional file 6.

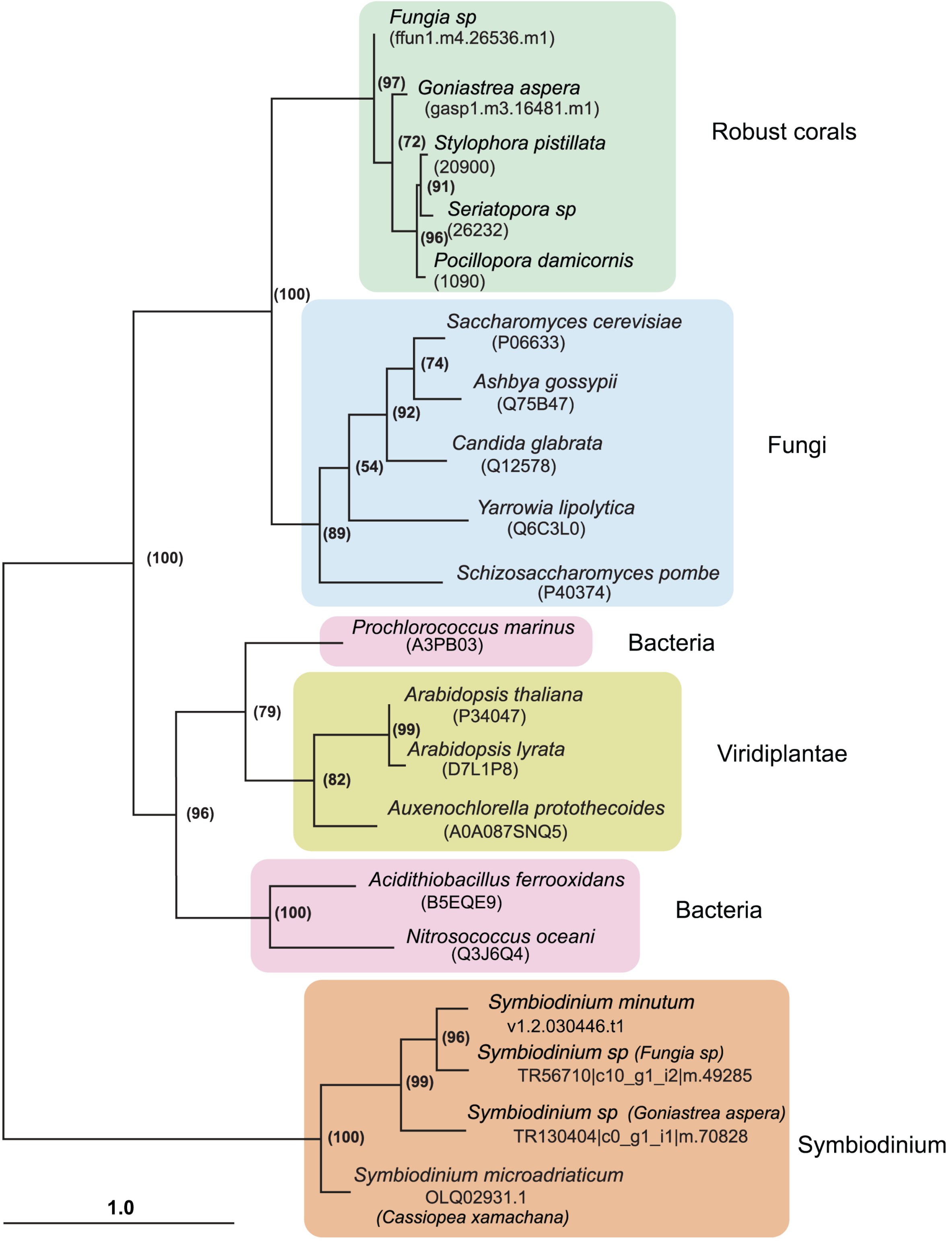

Figure S11: Phylogenetic tree of Imidazoleglycerol-phosphate dehydratase (K01693) proteins. IQ-TREE was used to generate the unrooted tree shown. Automatic model selection chose LG+R4 as the best model and 1000 UFBoot replicates were used to generate support values shown as node labels. The tree is displayed as rooted at the midpoint. Sequences on which the analyses are based are summarised in additional file 2:Table S21 and provided in Additional file 7. In general, the *Symbiodinium* proteins are significantly longer than other sequences, and typically contain two IGPD domains.

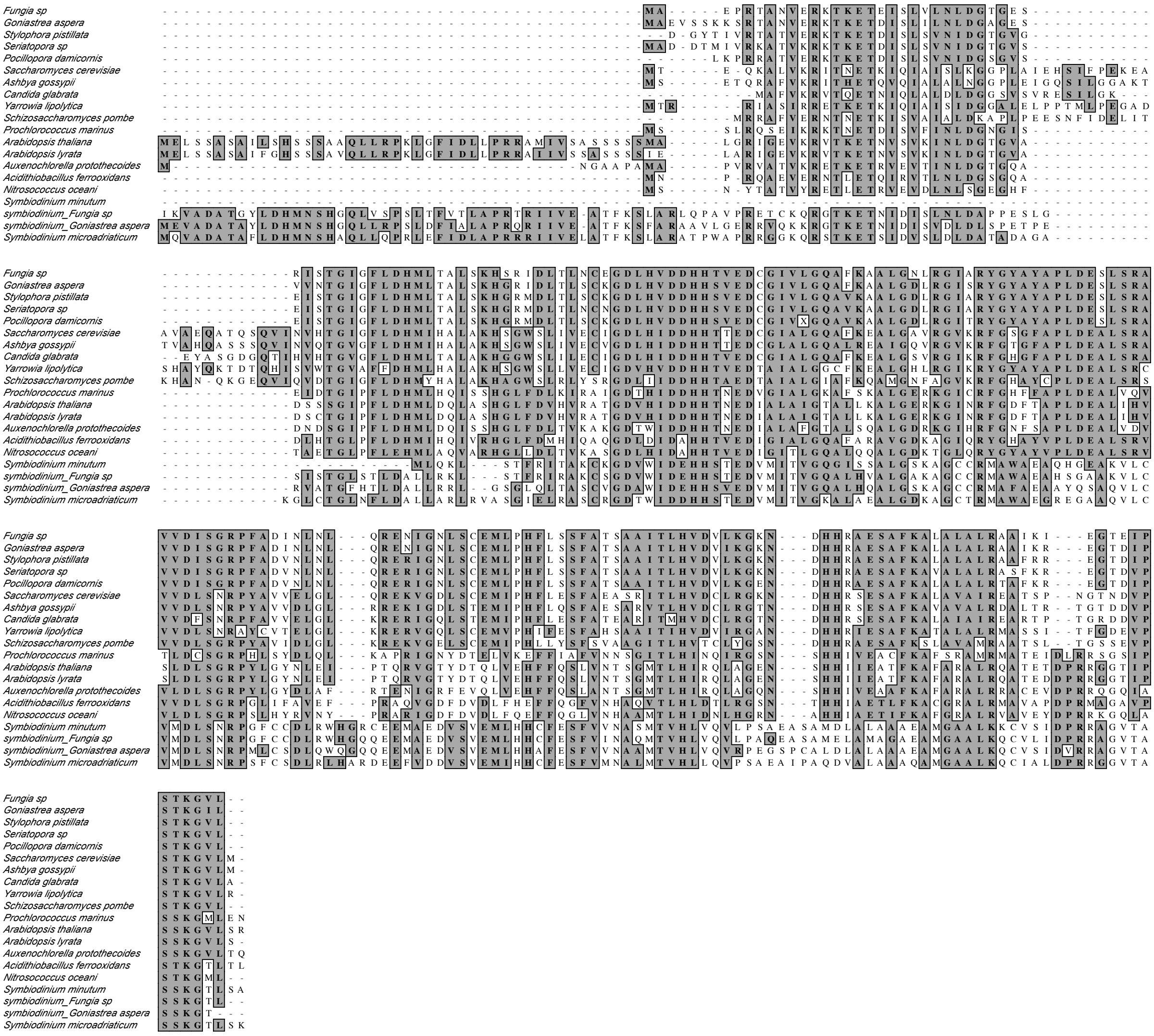

Figure S12: Alignment of Imidazoleglycerol-phosphate dehydratase (K01693) protein sequences. Poorly aligned columns with less than 20% alignable sequences were removed. The alignment shown was used to generate the phylogenetic tree Figure S11. In the case of *Symbiodinium* strains, the host species are indicated after the underscore. Sequences are summarised in Additional file 2: Table S21. Full length of annotated protein sequences are provided in Additional file 7.
